# Study on the mechanism of Shenkang injection in the treatment of chronic renal failure based on the strategy of "Network pharmacology—Molecular docking—Key target validation"

**DOI:** 10.1371/journal.pone.0291621

**Published:** 2023-10-05

**Authors:** Lin Zhou, Xiaohui Wang, Zhi Sun, Xiaoyue Bao, Lianping Xue, Zhanmei Xu, Pengfei Dong, Jinlan Xia

**Affiliations:** 1 School of Minerals Processing and Bioengineering, Central South University, Changsha, China; 2 Department of Pharmacy, the First Affiliated Hospital of Zhengzhou University, Zhengzhou, China; 3 Department of Ultrasound, the First Affiliated Hospital of Zhengzhou University, Zhengzhou, China; 4 Department of Chinese Medicine, the Second Affiliated Hospital of Zhengzhou University, Zhengzhou, China; Zagazig University, EGYPT

## Abstract

**Objective:**

To explore the potential mechanism of Shenkang injection (SKI) in the treatment of chronic renal failure based on network pharmacology and molecular docking technology, and to verify the core targets and key pathways by using the renal failure model.

**Methods:**

The active components and targets of Shenkang injection were retrieved by TCMSP database, and the disease related targets were obtained by OMIM, GeneCards and other databases. Then, the intersection was obtained, and were imported into String database for PPI analysis. After further screening of core targets, GO and KEGG analysis were performed. Autodock software was used to predict the molecular docking and binding ability of the selected active ingredients and core targets. Chronic renal failure (CRF) model was established by adenine induction in rats, and the pathological observation of renal tissues was conducted. Meanwhile, the effects of Shenkang injection and its active components on core targets and pathways of renal tissues were verified.

**Results:**

The results of network pharmacology showed that the main components of Shenkang injection might be hydroxysafflor yellow A (HSYA)、tanshinol、rheum emodin、Astragaloside IV. Through enrichment analysis of core targets, it was found that Shenkang injection may play an anti-chronic renal failure effect through PI3K-Akt signaling pathway. Molecular docking results showed that the above pharmacodynamic components had strong binding ability with the target proteins PI3K and Akt. The results of animal experiments showed that renal function indexes of Shenkang injection group and pharmacodynamic component group were significantly improved compared with model group. HE staining results showed that the pathological status of the kidney was significantly improved in SKI and pharmacodynamic component treatment groups. Immunohistochemical results showed that the renal fibrosis status was significantly reduced in SKI and pharmacodynamic component treatment groups. q-RTPCR and WB results showed that the expression levels of PI3K and Akt were significantly decreased in the treatment groups (P< 0.05).

**Conclusions:**

Shenkang injection may inhibit PI3K-Akt signaling pathway to play an anti-chronic renal failure role through the pharmacodynamic component hydroxysafflor yellow A (HSYA), tanshinol, rheum emodin, Astragaloside IV.

## Introduction

Chronic renal failure (CRF) refers to the clinical syndrome of chronic progressive renal parenchymal damage caused by various reasons, resulting in obvious atrophy of the kidney and inability to maintain basic functions, which is mainly manifested by the retention of metabolic products, imbalance of water, electrolyte and acid-base balance, and involvement of various systems [1, 2]. Clinical treatment mainly relies on the correction of acidosis, water and electrolyte disorders, the treatment of hypertension, the use of antibiotics to prevent infection, and the development of dialysis and kidney transplantation in later stages [3]. However, none of these treatments can cure the disease effectively at present, and the treatment level needs to be further improved.

Traditional Chinese Medicine is a cultural treasure of the Chinese nation and the crystallization of the wisdom of the working people for thousands of years [4]. It has made great contributions to ensuring people’s lives and safety. At the same time, traditional Chinese medicine has attracted more and more attention and recognition from many clinicians and experts because of its high efficiency and low toxicity [5]. Traditional Chinese medicine believes that kidney disease belongs to *ben xu biao shi*, its pathogenesis is a variety of chronic diseases for a long time not to heal, leading to *du*, *yu*, *xie*, *shi* accumulation and obstruction of meridians, viscera damage, resulting in spleen and kidney deficiency [6]. Shenkang injection is a traditional Chinese medicine injection made of rhubarb, salvia miltiorrhiza, safflower and astragalus for chronic renal failure under the guidance of traditional Chinese medicine theory, in which Astragalus and rhubarb are used together to “*fu zheng qu xie”* to protect kidney function [7]. The combination of salvia miltiorrhiza and safflower has the effect of promoting blood circulation, eliminating blood stasis and relieving pain, and can significantly improve microcirculation disorders, regulate the hypercoagulability of patients with renal failure, and delay the development of chronic renal failure. Therefore, Shenkang injection is especially suitable for chronic renal failure syndrome of “*shi zhuo xue yu*” [8]. We have conducted a comprehensive study on the pharmacodynamic component profile of Shenkang injection, a total of 90 compounds from SK injection were identified, including phenol and phenolic acids, flavonoids, anthraquinones, phenanthrenequinones and saponins, detailed chemical composition of Shenkang Injection was shown in S1 Table [9].

Although Shenkang injection has a good therapeutic effect on chronic renal failure, its therapeutic mechanism is still unclear, which not only hinders the clinical promotion and use of Shenkang injection, but also is not conducive to the development and use of related target drugs [10]. Therefore, in this study, network pharmacology was used to initially explore the main pharmacodynamic components and possible mechanisms of Shenkang injection in the treatment of chronic renal failure, molecular docking technology was used to confirm the docking stability between pharmacodynamic components and core targets, and experiments were conducted to verify the predicted results of network pharmacology and molecular docking, so as to reveal the mechanism of Shenkang injection in the treatment of chronic renal failure from a scientific perspective. This research will provide a scientific reference for future research on TCM treatment of complex diseases.

## Materials

### Main network database and software

TCMSP, Pubchem, Swiss Target Prediction, PharMapper Database; Online Mendelian Inheritance in Man (OMIM), GeneCards, Therapeutic Target Database (TTD), Genetic Association Database (GAD), PharmGkb disease target database; Uniprot, Research Collaboratory for Structural Bioinformatics Protein Data Bank (RCSBPDB) and other target information databases such as Database for Annotation, Visualization and Integrated Discovery (DAVID), String, Cytoscape3.9.1, Autodock, pymol, OpenBabel-3.1.1.

### Experimental drug

Shenkang Injection was purchased from Xi’an Century Shengkang Pharmaceutical Co., LTD.,. Hydroxyl safflower Yellow pigment A, Danshensu, emodin and astragaloside IV were purchased from Chengdu Durst Biotechnology Co., LTD.

### Animal

40 SD rats with body weight of 200-220g were purchased from Henan Experimental Animal Center. Animal Production License No.: SCXK(Yu)2017-0001; Before the test, the animals were kept in Specific pathogen Free (SPF) with standard rodent food and water, light and dark cycles every 12 hours at constant room temperature. The experiment was divided into control group, model group, Shenkang injection group, mixed drug group.

### Reagents

RIPA Lysate (batch number: R0010), BCA Protein Concentration Determination Kit (Batch number: PC0020), SDS-PAGE Protein Sample Loading Buffer (Batch number: P1200), was purchased from Beijing Solarbio Technology Co., LTD. Antibodies p-PI3K, PI3K, p-Akt, Akt (batch number:YT0180) and GAPDH (batch number:YM3215), and other reagents such as urinary creatinine were purchased from Wuhan service Biotechnology Co., LTD.

### Instrument

041BR69450 electrophoresis apparatus, VE-180 vertical plate electrophoresis device was purchased from Bole Life Medical Products (Shanghai) Co., LTD. GeLView 6000plus gel Imaging System (Guangzhou Bolueng Biotechnology Co., LTD.,), ASP200S automatic dehydrator, DM3000 in-place Fluorescence microscope, RM2235 paraffin slicer and 610020-9Q chemiluminescence Instrument (Shanghai Qinxiang Scientific Instrument Co., LTD.); Model C16000 automatic biochemical analyzer (Abbott Co., LTD.).

## Methods

### Screening of active ingredients and targets of Shenkang injection

Using safflower, Salvia miltiorrhiza, rhubarb and Astragalus as key words, the active constituents and related targets were retrieved from TCMSP. This research searches the active ingredient and target for drug-like DL(Drug Likeness)≥0.18 & oral bioavailability (OB)>30 in TCMSP, and searches the Chinese pharmacopoeia for the active ingredient to supplement the likeness. The SMILES string obtained by Pubchem was guided by SwissTargetPrediction platform, and the attribute was set as "homo sapiens", and all prediction target protein targets with Probability>0 were selected as effective prediction targets for this component. The component that failed to obtain the target from SwissTargetPrediction was imported from 2D structure obtained from Pubchem into PharMapper to obtain the target, and the attribute was set as "fit>2, Human".

### Screening of chronic renal failure related targets

Through OMIM, GeneCards, TTD, GAD, PharmGkb database, "chronic renal failure, Chronic renal impairment, chronic renal insufficiency, chronic kidney failure" was searched for the search term, and the target related to chronic kidney failure was obtained after the retrieval results were integrated to remove redundancy.

### Screening of potential targets for Shenkang Injection in the treatment of chronic renal failure

The intersection of the active ingredient target of Shenkang Injection and the target of chronic renal failure was taken as the potential target of Shenkang injection in the treatment of chronic renal failure from the Venn diagram.

### Construction of PPI network and visualization network

Cytoscape(3.9.1) was applied to visualize the interactions between proteins based on the human String database of the above potential targets, taking Homo sapiens and high confidence ≥ 0.9 as the screening conditions. Order the interactions according to network medium value, mediality and closeness to screening core targets.

### Enrichment analysis of GO biological processes and KEGG pathway

Related biological processes and action pathways of the selected core target genes were further analyzed in the Human DAVID database. The enrichment results were sorted from small to large (P<0.05). The top 10 pathways were selected for GO analysis, and the top 20 pathways were selected for KEGG analysis and display. KEGG database was used for annotation and pathway map analysis.

### Network diagram of "Drug ingredient—target -pathway"

KNIME software was used to obtain chemical components corresponding to the first 20 pathways related targets, and the corresponding relationship between active component and pathway related targets and pathway was obtained. The network diagram of "Drug component, target and pathway" was constructed by introducing Cytoscape, and the main active components were screened according to the degree value.

### Molecular docking to predict the binding ability of core components and target of Shenkang injection

Autodock software was used to verify the molecular docking between the top 4core targets and the corresponding active ingredients. The three-dimensional structure of the target protein was obtained from RCSB PDB protein structure database and saved in pdb format. The three-dimensional structure of the compound was exported from Pubchem database in SDF format and converted into MOL2 format in OpenBabel-3.1.1. AutoDock Tools was used to dehydrate the protein receptors, calculate the charge, and store them in pdbqt format. Then the plug-in Autodock was used to obtain the active pocket for molecular docking. Finally, pymol was used for visualization analysis.

### Study on optimization of pharmacodynamic ingredient composition ratio

The results of serum creatinine after different pharmacodynamic components intervention in renal failure rat model were analyzed in order to find the optimal composition. Orthogonal test was used, with 3 concentration levels for each component factor, 10% glucose injection was used as the solvent, and the changes of serum creatinine indexes in rats with chronic renal failure were observed by caudal intravenous injection at the dose of 0.9 mL/100g. The orthogonal table L9(3^4^) was designed, Range analysis method was used to calculate the data. T = $\sum \frac{n}{i=1}Xi$, R = *max*{T1,T2,T3}-*min*{T1,T2,T3},T is the sum of all indexes, and Ti is the sum of indexes corresponding to the level number i(i = 1,2,3) in any column. R is the range, and reflects the difference between the maximum value and the minimum value of the test index at each level of the factors in any column. The R value reflects the change amplitude of the test index when the level of each column of factors changes. The larger the value of R, the greater the influence of this factor on the test index, and therefore the more important it is. The primary and secondary factors can be judged according to the magnitude of R-value, and the optimal level combination of all factors can be obtained by using the optimal level and the order of the primary and secondary, namely the optimal combination.

### Effect and mechanism of Shenkang injection and its core pharmacodynamic components in treating chronic renal failure

The animals were divided into control group, model group, Shenkang injection group and mixed drug group by random number table method. Model preparation methods: 40 male SD rats, weighing 200–220 g, were randomly divided into 4groups, with 10 rats in each group after 7 days of adaptive feeding in an SPF thermostatic animal laboratory. Adenine was dispersed in sodium carboxymethyl cellulose solution. Except for the normal group, the other three groups were given continuous gavage of 250 mg·(kg·d)^-1^ to establish renal failure model group [11]. The detailed method was: the rats were given adenine suspension by gavage at the dose of 250 mg·(kg·d)^-1^ body weight, once a day for the first 12 days, and once every other day from the 13th day, the whole process lasted 24 days (administration at 9:00 am).

Shenkang injection group was injected with 9 mL·(kg·d)^-1^ Shenkang injection through the tail vein. The mixed drug group were injected with the same amount of mixed drugs through the tail vein, and the control group and model group were injected with the same amount of normal saline for 7 consecutive days. Animal Ethics Review Approval Institutions & Number: Ethics Committee of the First Affiliated Hospital of Zhengzhou University (2023-KY-0543). The informed consent statement was not applicable to this study, because this study was conducted on animals.

### Biochemical index detection

After the last administration, water was not allowed for 24 h after fasting. Animals were anesthetized by ether inhalation and euthanized. Blood was collected from abdominal aorta and the levels of serum creatinine (Scr), urea nitrogen (BUN), uric acid (UA) and electrolyte [K^+^, Na^+^, Ca^2+^ and P^3+^] in each group were detected.

### Renal pathological observation

The kidneys of rats were fixed with 10% neutral formaldehyde fixation solution, dehydrated and embedded, sliced, dewaxed to water, stained with HE, and the pathological changes of the kidneys of each group were observed under microscope. Pathological evaluation criteria was shown in S2 Table.

### Expression of α-SMA and E-cadherin in renal tissue was detected by immunohistochemistry

Kidney tissue was dephosphorized with xylene, rehydrated with gradient ethanol, inactivated endogenous peroxidase with 3% hydrogen peroxide, and heat recovery antigen with 0.1 mol/L citrate buffer. PBS solution containing 10% goat serum was added and incubated at room temperature for 30 min to block the binding of non-specific antibodies. α-SMA and E-cadherin antibodies were added and incubated overnight at 4°C. After rinsed with PBS solution, goat anti-rabbit antibody labeled by HRP was added. DAB was used for color development, stained with hematoxylin, observed under microscope and photographed.

### The mRNA expression of PI3K and Akt in renal tissues was detected by qRT-PCR

According to the product description, TRIzol extracted total RNA from kidney tissue, and Servicebio^®^RT First Strand cDNA Synthesis Kit synthesized cDNA. 2×SYBR Green qPCR Master Mix was used for quantitative detection of PI3K, Akt and GAPDH genes. Primer sequence: GAPDH: Forward direction: GGAAGCTTGTCATCAATGGAAATC, Reversed direction: TGATGACCCTTTTGGCTCCC; PI3K: Forward direction: AGAAAGCACTGACAAATCAAAGG, Reversed direction: ACAGGCACGGCAGTAGGAC; Akt: Forward direction: TGAACGACGTAGCCATGTGA, Reversed direction: GCAGCGGATGATGAAGTGT;

ΔΔCT method was used for result processing: A = CT_Target gene after drug intervention−_CT_Reference gene after drug intervention_, B = CT_Target gene without drug intervention−_CT_Reference gene without drug intervention_, K = A-B, Relative expression level = 2^-K^。

### The expression of PI3K-Akt signaling pathway related proteins in renal tissues was detected by western blotting

Kidney tissues of each group were collected and homogenized with RIPA lysate to extract protein. The protein samples were transferred to PVDF membrane by 10% sodium dodecyl sulfate-polyacrylamide gel electrophoresis. After sealing, p-PI3K, PI3K, p-Akt, Akt and GAPDH antibodies were added, respectively, and incubated at 4°C overnight. HRP labeled goat anti-rabbit antibody was added, incubated for 2 h, and the developer was added for color development. ECL chemiluminescence instrument was used for development.

### Statistical analysis

SPSS 22.0 software was used for data statistics, which were expressed as x±s and tested by one-way analysis of variance. *P*<0.05 was considered statistically significant.

## Results

### Effective components and corresponding targets of Shenkang injection in the treatment of chronic renal failure

**Fig 1:**
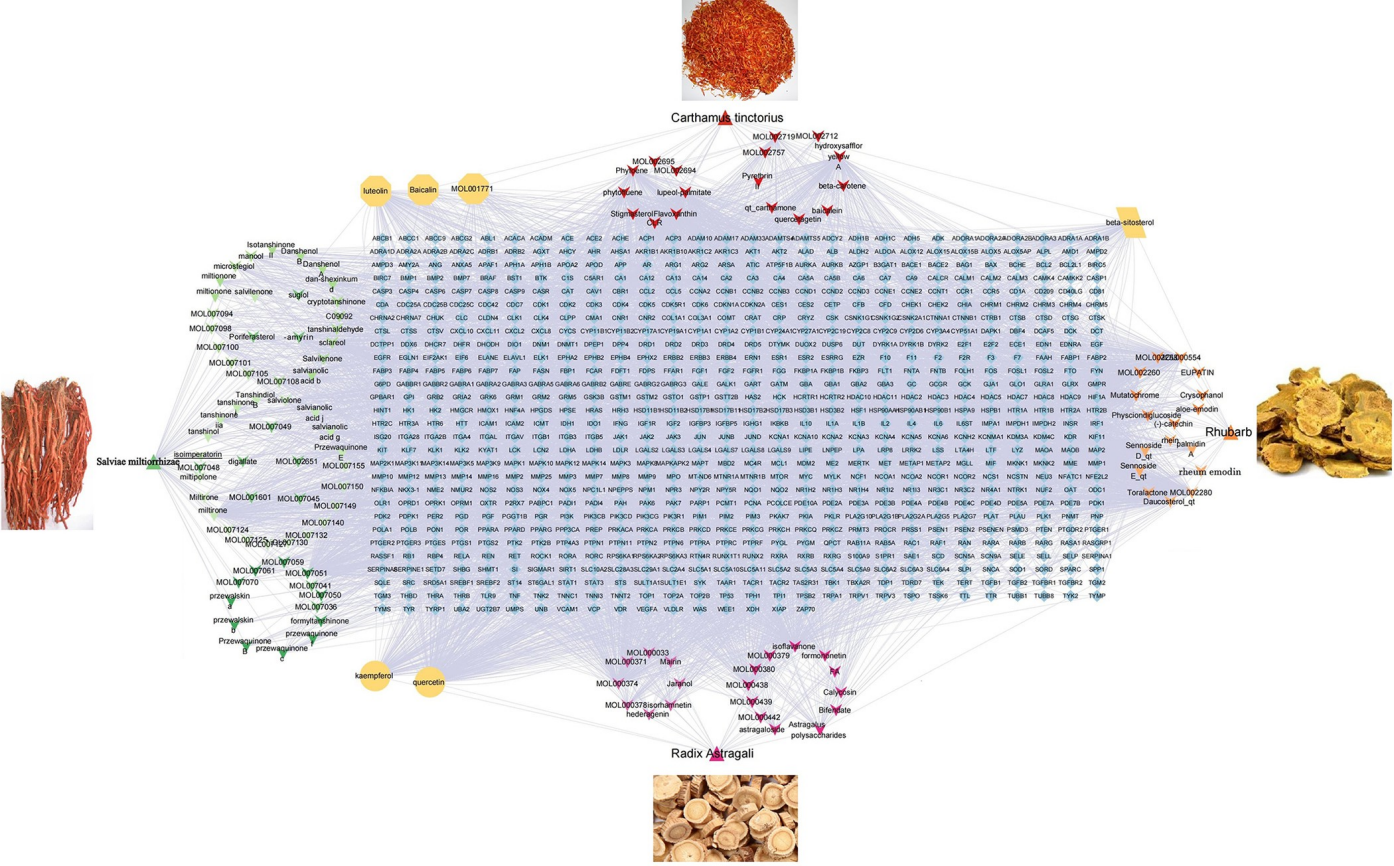
Effective components and corresponding targets of Shenkang injection in the treatment of chronic renal failure (Annotation: The upright triangle represents the medicine, the upside down triangle represents the compound, the diamond represents the target).

In this study, 23 compounds from safflower [12], 67 compounds from salvia miltiorrhiza [9], 18 compounds from rhubarb [13] and 22 compounds [14] from astragalus in Shenkang Injection were obtained from TCMSP, SwissTarget Prediction and PharMapper database screening, and 802 potential targets were obtained after integrated deweighting. Results, a total of 929 points and 6139 edges were visualized using Cytoscape, as shown in Fig 1.

### Screening and intersection of targets related to chronic renal failure

A total of 253, 811, 412 and 211 targets related to chronic renal failure(According to the International Classification of Diseases ICD-10, the chronic renal failure ID was N18.9) were retrieved from OMIM, GeneCards, TTD, and PharmGkb disease gene databases, respectively, and 1691 disease targets were obtained after integration. The intersection targets of active ingredients and chronic renal failure related targets were selected, and 491 overlapping genes were obtained, accounting for 24.5% of the total number of genes, which were potential targets of Shenkang injection in the treatment of chronic renal failure.

### Analysis of potential target protein interaction (PPI) network

**Fig 2:**
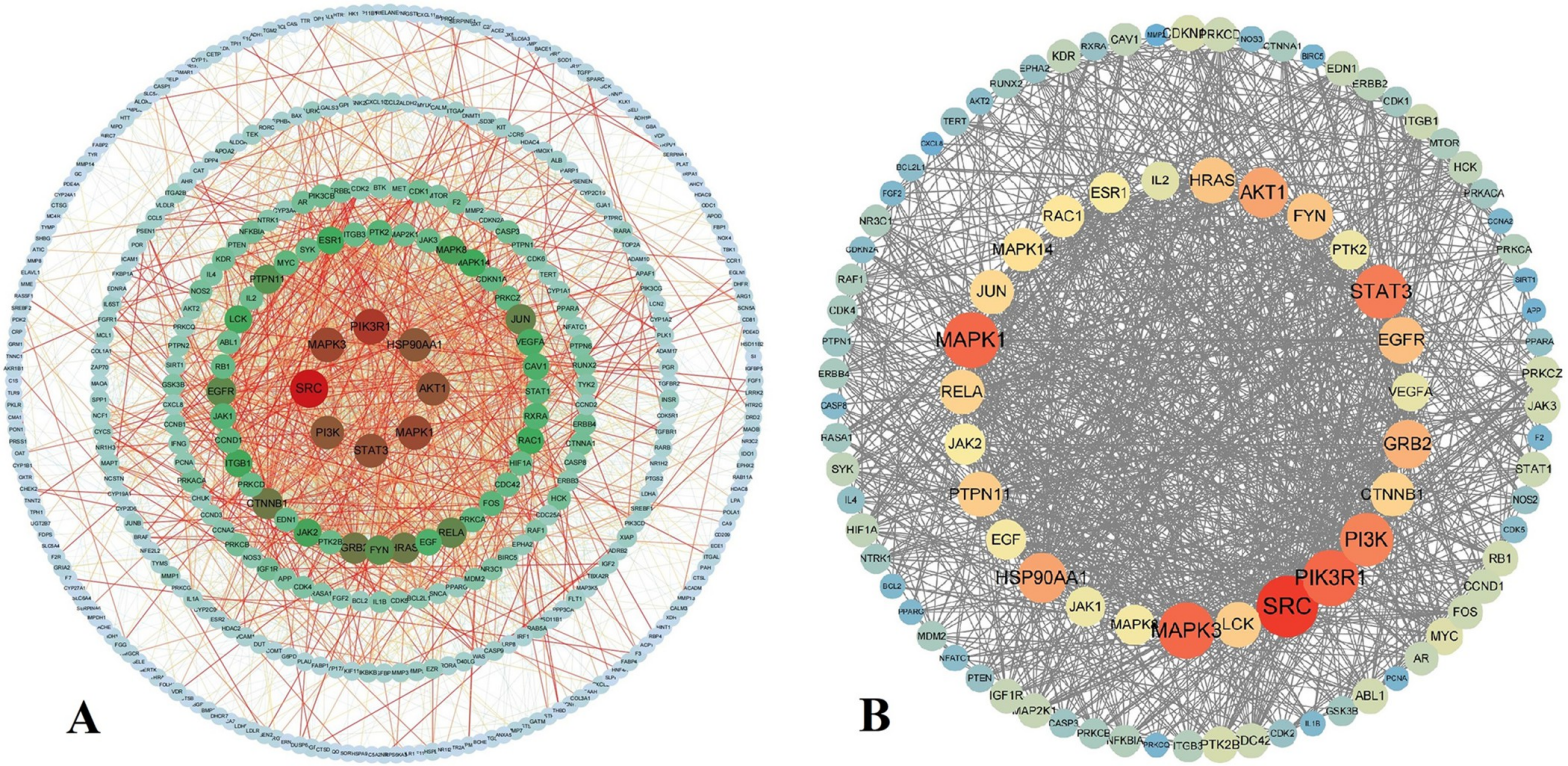
Potential target protein interaction (PPI) network (Annotation: The smaller circle represents the target, and the larger the circle, the more important the target).

491 potential targets were imported into the String database for PPI network analysis. The results were visualized using Cytoscape. The PPI network contains 422 targets and 2573 edges, as shown in Fig 2A. A total of 97 core targets were screened with degree value ≥2 times median, medianness and compactness ≥ median, in which the darker the color, the greater the degree value, as shown in Fig 2B.

### GO results

**Fig 3:**
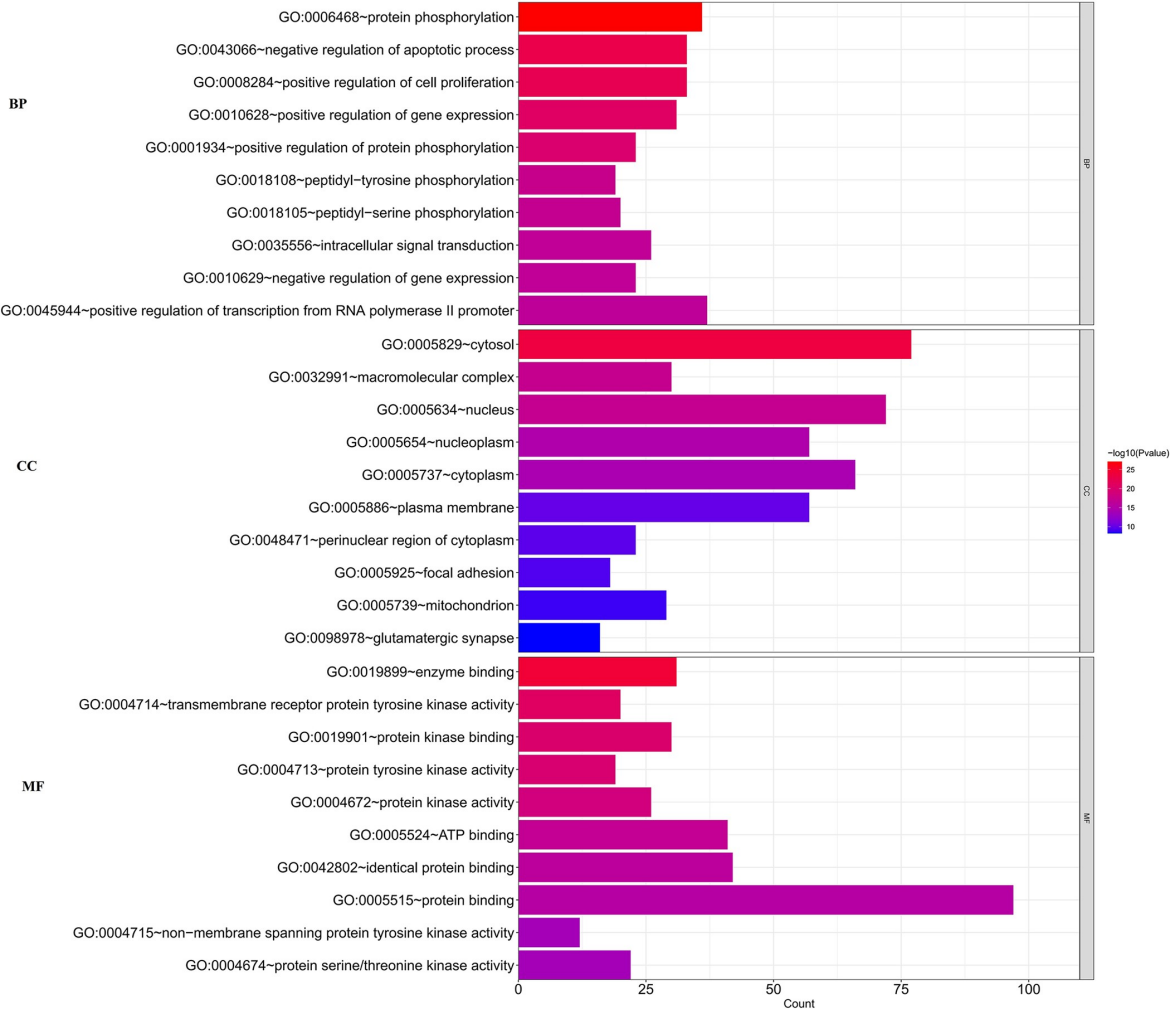
The results of GO functional enrichment analysis (Annotation: The higher the value, the greater the importance).

GO functional enrichment analysis was conducted on the above targets through the DAVID database, and 835 significant GO items were obtained, including 643 biological process (BP) items, 72 cell composition (CC) items and 120 molecular function (MF) items involved. The top 10 items were listed, as shown in Fig 3.

### KEGG results

**Fig 4:**
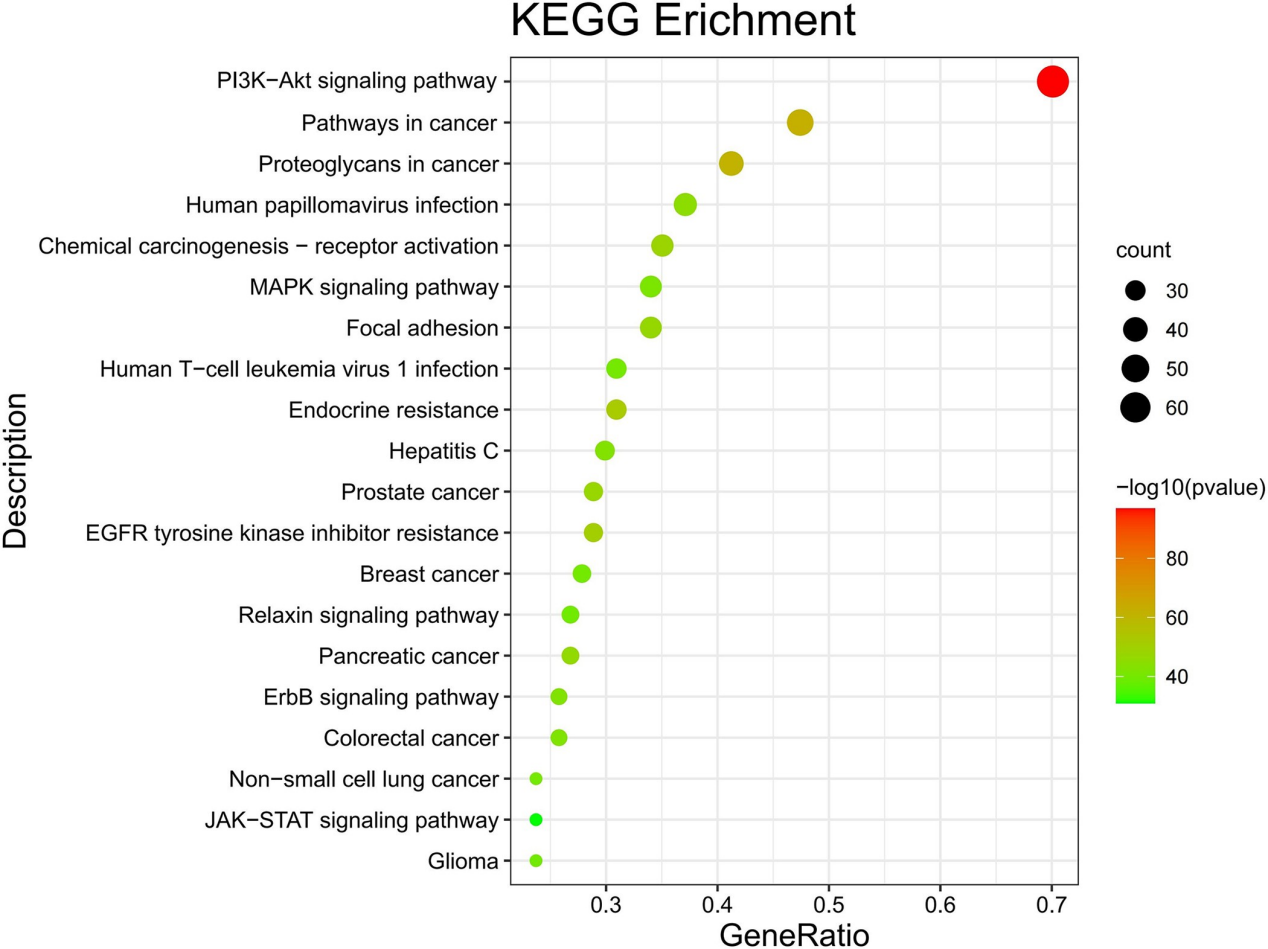
The results of KEGG enrichment analysis (Annotation: Circles represent each pathway, and the redder the color, the larger the circle, and the more important it is).

Select the pathway with the top 20 enriched genes plotted by (https://www.bioinformatics.com.cn, a free online platform for data analysis and visualization.). Drawing the bubble diagram, as shown in Fig 4.

### Construction of the network of "Drug ingredient—target—pathway"

**Fig 5:**
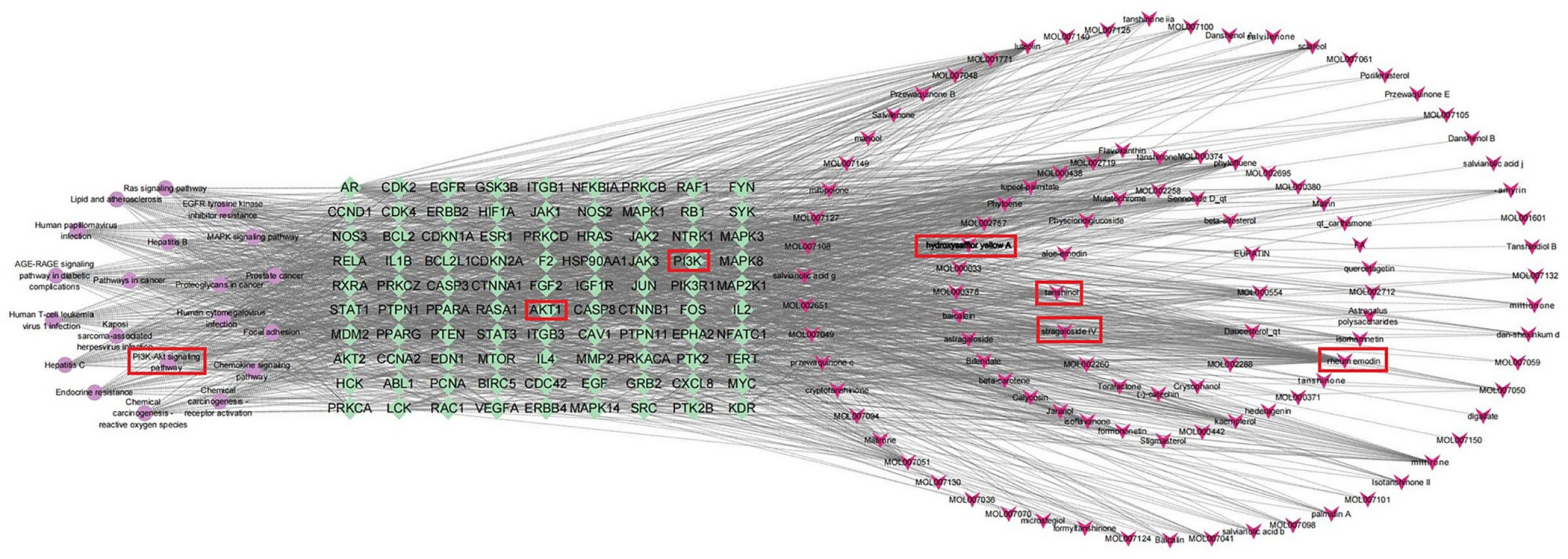
The network of "Drug ingredient—target—pathway" (Annotation: Circles represent pathways, diamonds represent targets, and triangles represent compounds).

The obtained components, targets, and pathways from the above network pharmacology studies will be imported into Cytoscape to construct the "drug ingredient-target-signaling pathway" network diagram, as shown in Fig 5. The network involved 217 nodes and 1197 edges, and the average degree value was 16. There were 20 active components with higher than the average degree value, among which the components with the highest degree value were HSYA, tanshinol, rheum emodin and Astragaloside IV. It is suggested that these ingredients may be the main active ingredients of Shenkang injection in the treatment of chronic renal failure, as shown in Fig 5 and S3 Table.

### Docking of molecules

**Fig 6:**
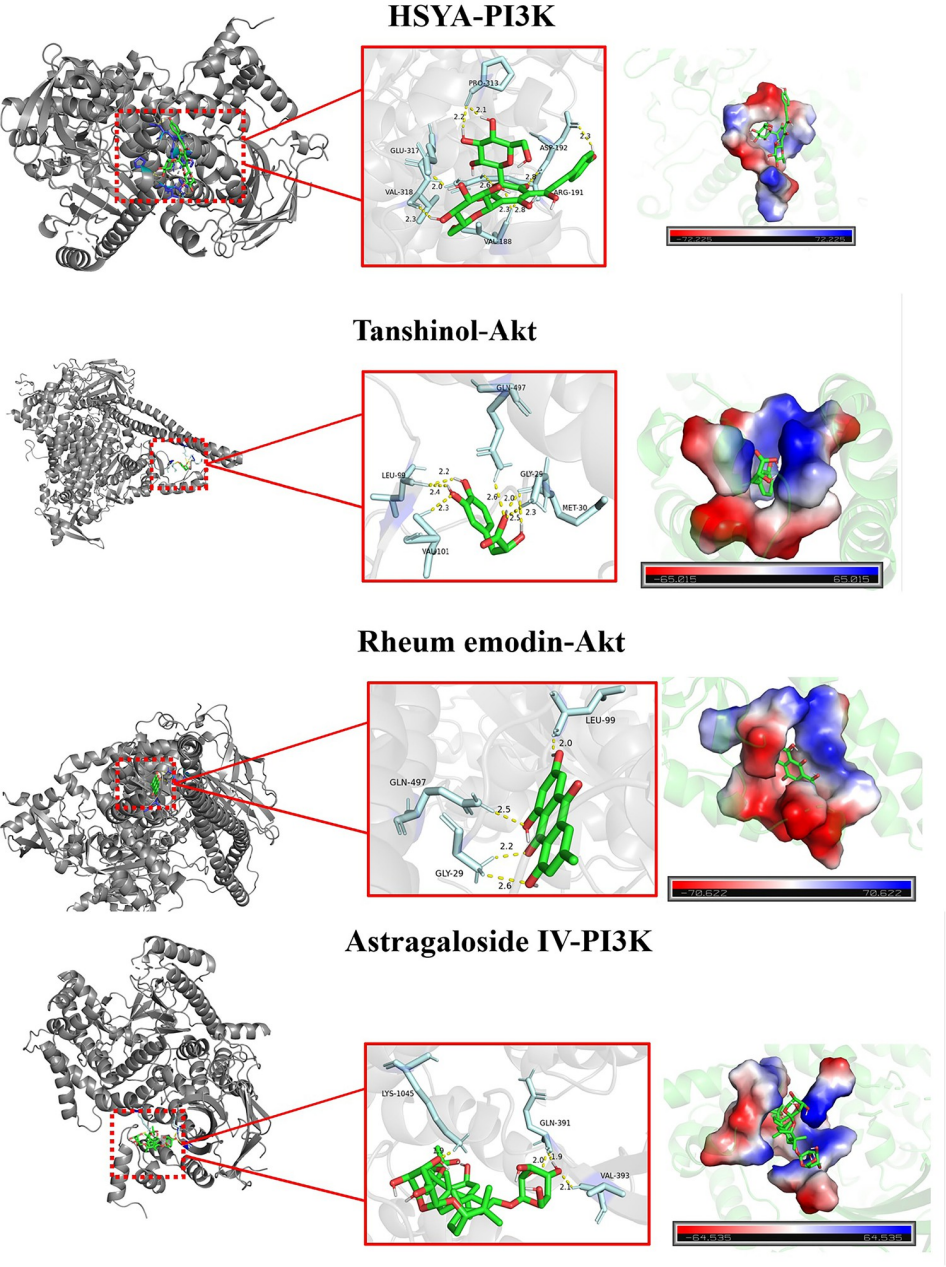
The results for docking of molecules (Annotation: Four components and their corresponding target docking process).

In order to verify the accuracy of the prediction results of network pharmacology, the core target PI3K and Akt in the top ranking signaling pathway were docked with the main active components HSYA, tanshinol, rheum emodin and Astragaloside IV. The smaller the binding energy between the target and the active component, the better. Binding energy <-5 kcal/mol, indicating that the two units have good binding activity, as shown in Fig 6 and S4 Table.

### Study on optimization of pharmacodynamic ingredient composition ratio

The data were calculated by range analysis method, as shown in Tables 1–3. According to the above range analysis results, the R-values of HSYA, tanshinol, rheum emodin and Astragaloside IV are 16.6, 39.9, 8.8 and 9.7, respectively. The results showed that the influence of ingredients on the efficacy of tanshinol>HSYA>Astragaloside IV> rheum emodin; According to the results of orthogonal test, the best pharmacodynamic component combination was No.6, which had the greatest decrease in serum creatinine and the best effect. From the aspect of factor level, the sum of HSYA factor 2 level, tanshinol factor 3 level, rheum emodin factor 1 level and Astragaloside IV factor 2 level are the largest. Therefore, A2B3C1D2 combination is the best. HSYA (0.2mg/mL), tanshinol (2.0mg/mL), rheum emodin (0.07mg/mL) and Astragaloside IV (0.06mg/mL) were the best combination of components.

**Table 1:** Level table of orthogonal test factors.

Level	HSYA	tanshinol	rheum emodin	Astragaloside IV
	mg/mL	mg/mL	mg/mL	mg/mL
1	0.1	0.5	0.07	0.03
2	0.2	1.0	0.14	0.06
3	0.4	2.0	0.28	0.12

**Table 2:** Results of orthogonal test data statistics.

No	HSYA	tanshinol	rheum emodin	Astragaloside IV	Creatinine level
1	1	1	1	1	11.3% ↓
2	1	2	2	2	22.3% ↓
3	1	3	3	3	23.2% ↓
4	2	1	2	3	15.0% ↓
5	2	2	3	1	24.7% ↓
6	2	3	1	2	33.7% ↓
7	3	1	3	2	15.0% ↓
8	3	2	1	3	25.4% ↓
9	3	3	2	1	24.3% ↓

**Table 3:** Range analysis results of each factor.

HSYA			tanshinol			rheum emodin			Astragaloside IV		
C	T	R	C	T	R	C	T	R	C	T	R
0.1	56.8	16.6	0.5	41.3	39.9	0.07	70.4	8.8	0.03	60.3	9.7
0.2	73.4		1	72.4		0.14	61.6		0.06	71.0	
0.4	64.7		2	81.2		0.28	62.9		0.12	63.6	

### Biochemical index detection results

**Fig 7:**
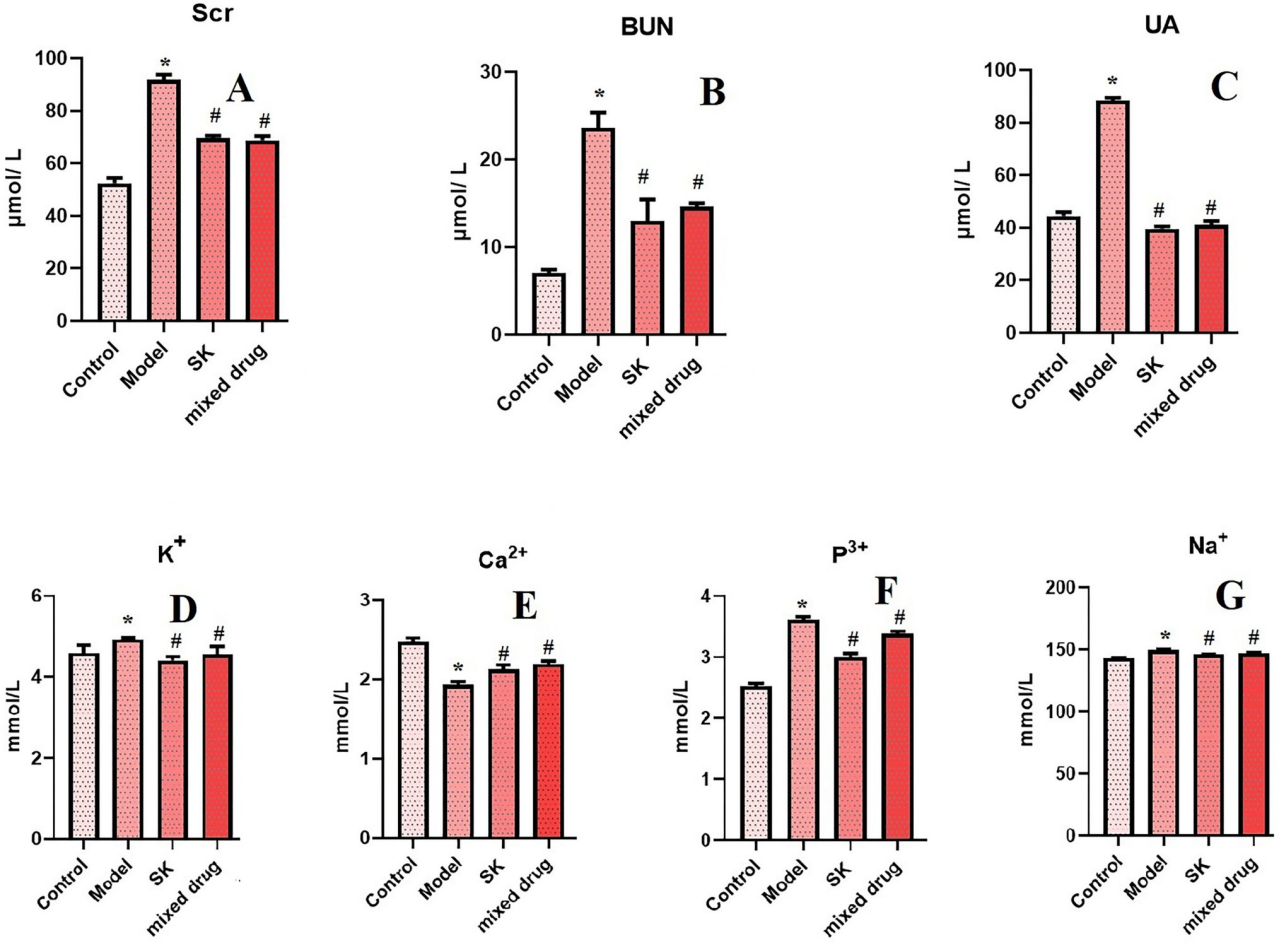
The results of biochemical index detection in different groups.

The results of biochemical indexes showed that compared with normal control group, the concentrations of Scr, BUN, UA, K^+^, P^3+^ and Na^+^ in serum of rats in model group increased, and the concentration of Ca^2+^ decreased with statistical significance (*P < 0.05). Compared with model group, Scr, BUN, UA, K^+^, P^3+^ and Na^+^ in serum of Shenkang injection group and mixed drug groups were significantly decreased, while Ca^2+^ concentration was increased, with statistical significance (^*#*^*P* < 0.05). The results showed that Shenkang injection and its components could significantly reduce the serum levels of Scr, BUN, UA, K^+^, P^3+^, Na^+^ and increase the serum levels of Ca^2+^ in model rats, as shown in Fig 7.

### Pathological observation of kidney in rats

**Fig 8:**
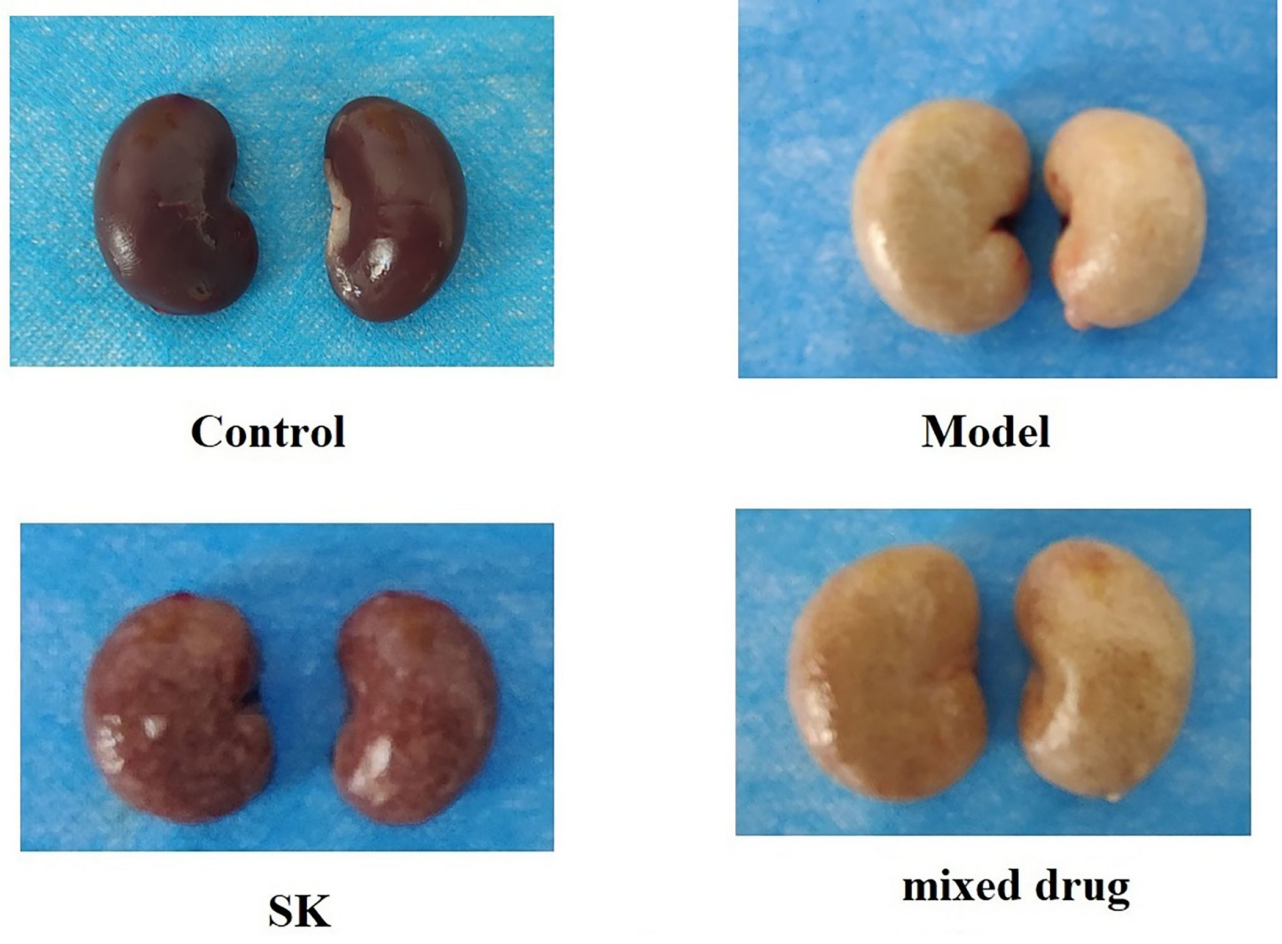
External morphology of kidney in different groups.

**Fig 9:**
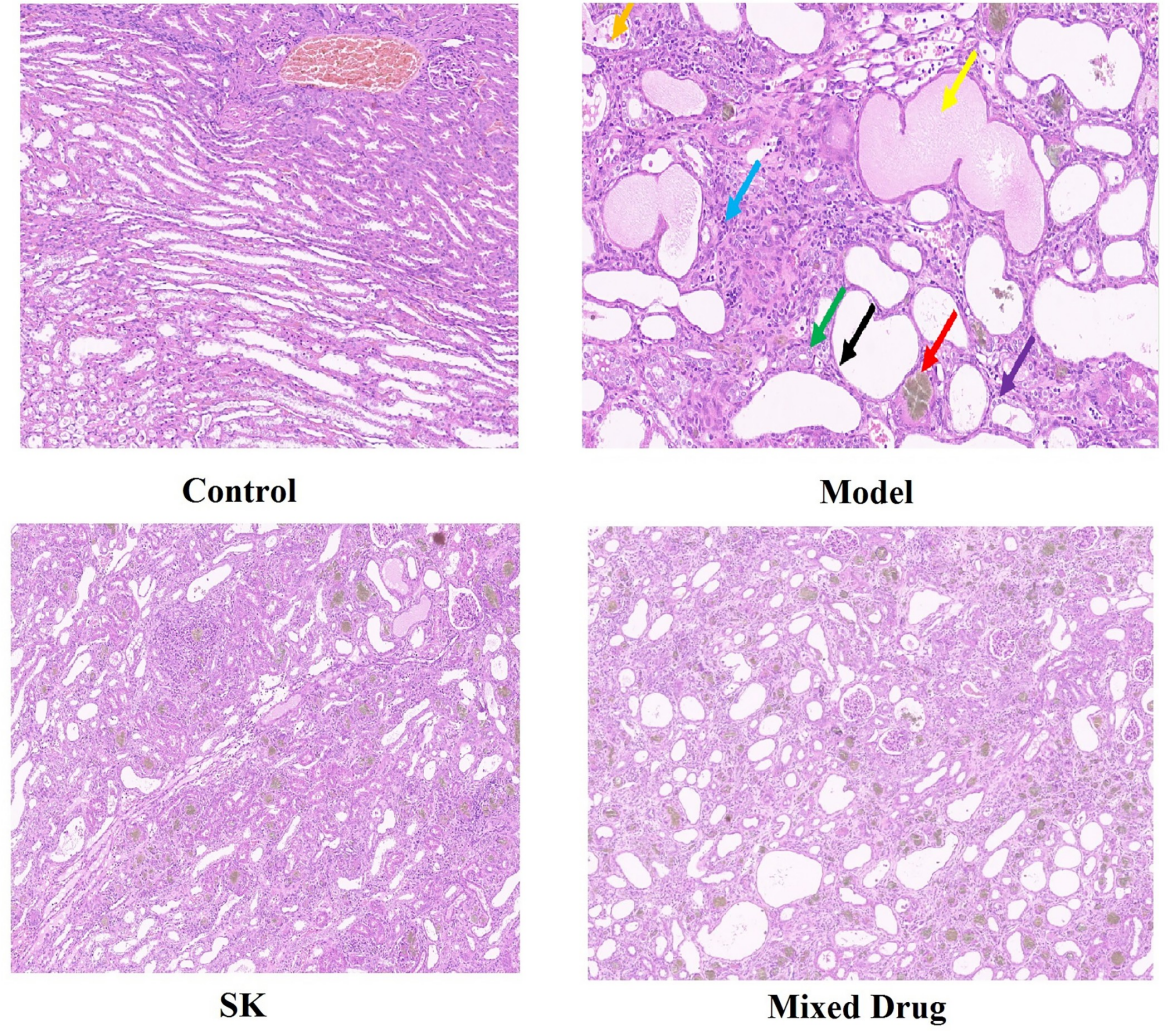
The HE staining results in different groups.

In the normal control group, the kidney was dark red in color and no significant histological changes were observed. HE staining showed no lesions in renal tubules, glomeruli, mesangial area width, mesangial matrix and interstitium. Compared with the normal control group, the kidney of model group was grayish white, HE staining showed that in the model group, a large number of renal tubules were dilated and epithelial cells were flattened (black arrow). A large number of renal tubular epithelial cells with watery degeneration, loose cytoplasm light staining (purple arrow) appeared. Diffuse connective tissue hyperplasia was seen in the intertubule interstitium with extensive lymphocyte infiltration (blue arrow), numerous tubule atrophy, lumen narrowing or disappearance (green arrow) appeared. A small number of protein tubes (yellow arrow) could be seen; urate crystals (red arrow) were observed in a large number of renal tubules. A small number of necrotic cells appeared in a few renal tubules (orange arrow). Compared with the model group, the renal interstitium of Shenkang injection group and the mixed drug group was significantly improved, with fewer inflammatory cells and thinner vascular walls, as shown in Figs 8 and 9. The results of relevant pathological scores were shown in Table 4.

**Table 4:** The results of pathological scores.

Group	hydropic degeneration	Renal tubule dilatation	Inflammatory cell infiltration	Protein tube type
Control	0	2	0	0
Model	3	4	4	2
SK	2	3	3	1
Mixed Drug	0	3	2	1

### Immunohistochemical results of renal tissue

**Fig 10:**
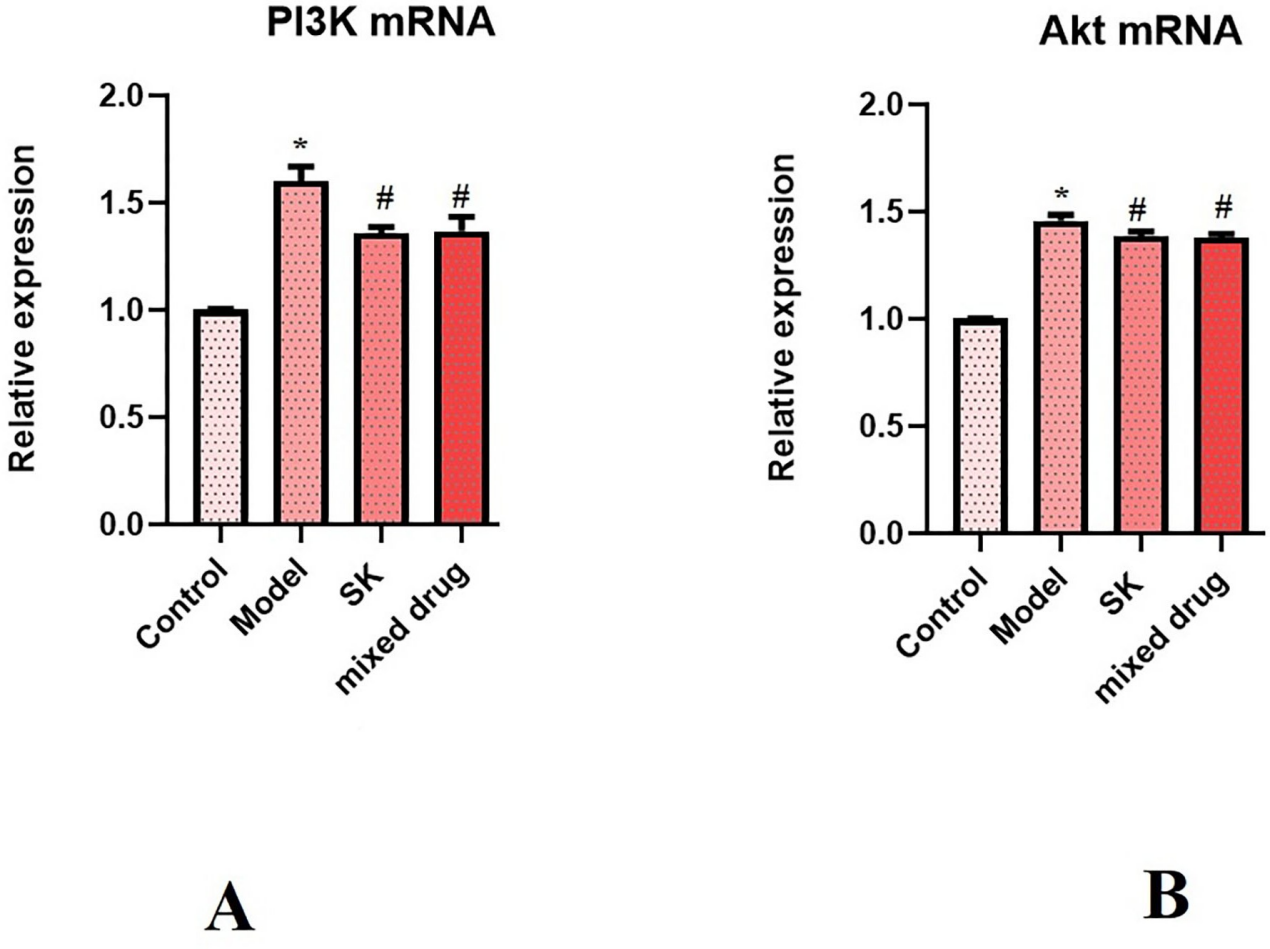
Immunohistochemical results of renal tissue in different groups.

Compared with the normal control group, the expression of E-cadherin in the kidney of model group was significantly decreased, and the expression of α-SMA protein was increased, indicating the occurrence of renal fibrosis. Compared with the model group, the expression of E-cadherin in Shenkang injection group and mixed drug group increased, and the expression of α-SMA protein decreased, indicating that the degree of fibrosis in the drug treatment group was relieved, shown in Fig 10.

### The mRNA expression of PI3K and Akt determined by qRT-PCR

**Fig 11:**
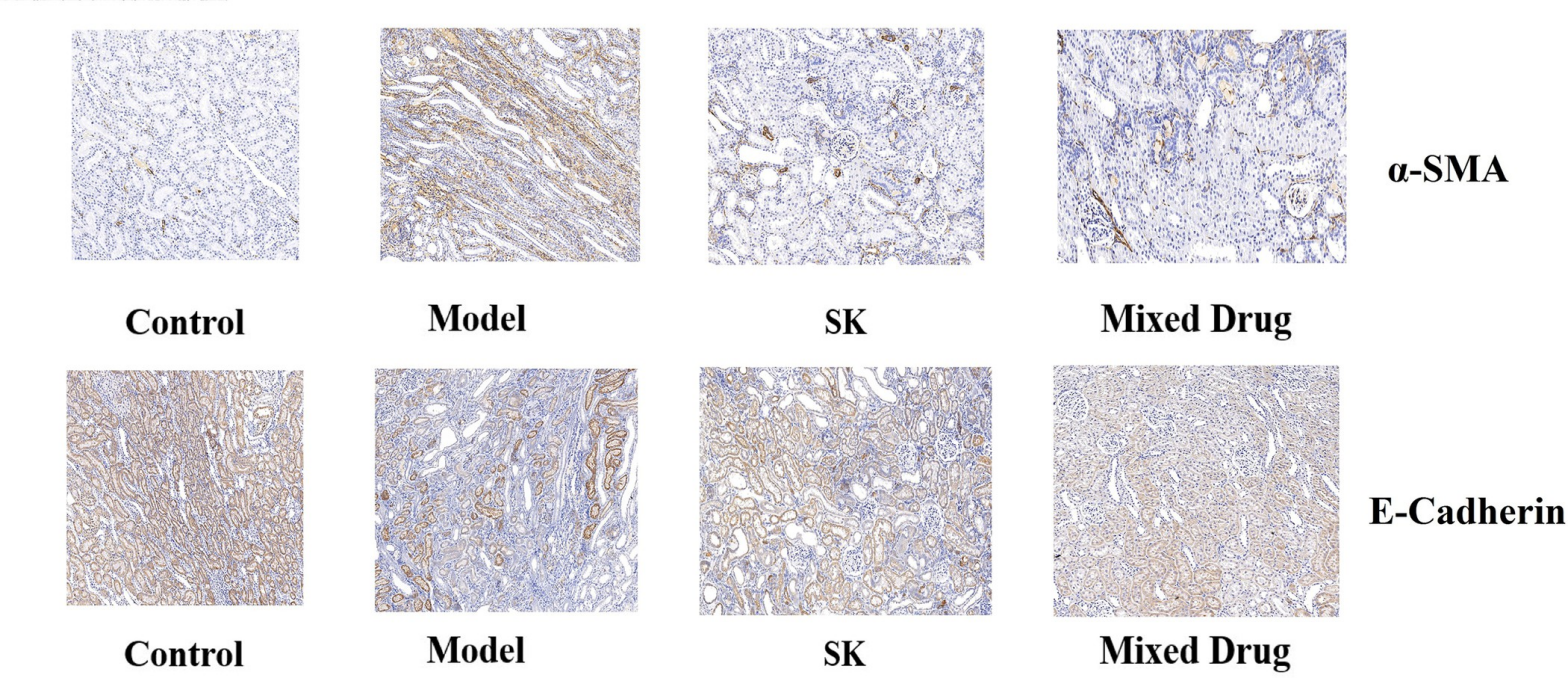
The mRNA expression of PI3K and Akt determined by qRT-PCR in different groups.

The results of qRT-PCR showed that compared with normal control group, the mRNA expression levels of PI3K and Akt in model group were significantly increased, with statistical significance (*P < 0.05). Compared with model group, the mRNA expression levels of PI3K and Akt in Shenkang injection group, mixed drug group were significantly decreased, with statistical significance (*#P* < 0.05). The results showed that Shenkang injection and its components could significantly reduce the expression levels of PI3K and Akt mRNA in the kidney, as shown in Fig 11.

### The protein expressions of PI3K and Akt determined by western blotting

**Fig 12:**
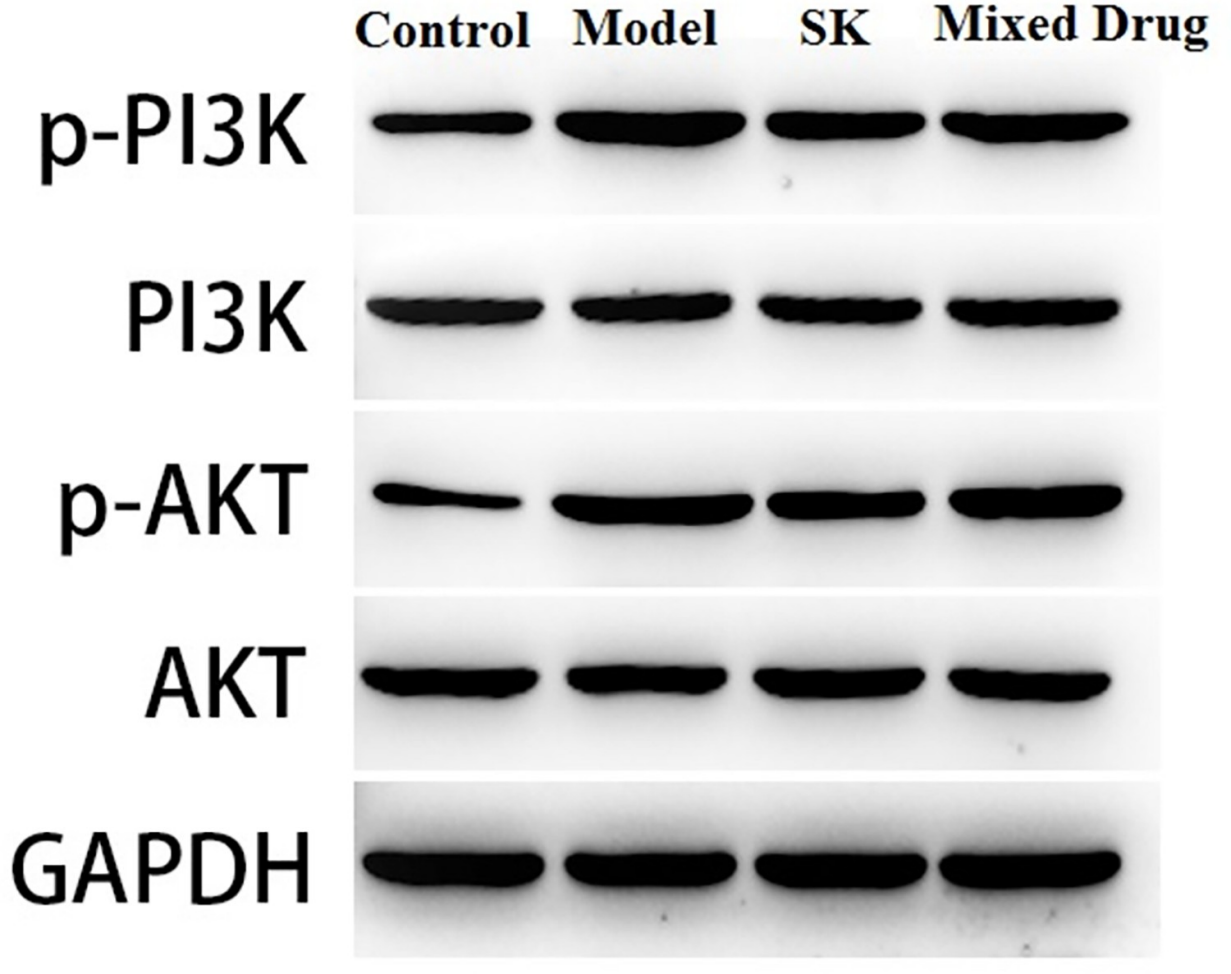
The protein expressions of PI3K and Akt were determined by western blotting in different groups.

WB results showed that compared with normal control group, the expression levels of P-PI3K/PI3K, P-Akt/Akt in model group were significantly increased, with statistical significance (*P < 0.05). Compared with model group, the expression levels of P-PI3K/PI3K, P-Akt/Akt in Shenkang injection group, mixed drug group were significantly decreased, with statistical significance (^*#*^*P* < 0.05). The results showed that Shenkang injection and its components could significantly reduce the expression levels of P-PI3K/PI3K, P-Akt/Akt in the kidney, as shown in Fig 12.

## Discussions

In this study, network pharmacology and molecular docking techniques were used to identify the key pharmacodynamic components and key targets of Shenkang injection in the treatment of chronic renal failure, and experimental techniques were used to verify the scientific results.

Renal injury leads to changes in renal structure and function, resulting in abnormal excretion of certain electrolytes, retention and loss of electrolyte ions, increase or decrease of serum electrolyte content, and can cause serious complications such as renal anemia, renal rickets and disorders of human function [15]. Water and electrolyte disorders are common in chronic renal failure, mainly manifested as high potassium, low calcium, high phosphorus, and mild water and sodium retention [16]. In this study, the level of electrolyte K^+^, P^3+^ and Na^+^ increased and the level of Ca^2+^ decreased in the model group of rats. However, Shenkang injection and its drug composition group could improve the electrolyte index, indicating that the kidney tissue damage was effectively alleviated and part of renal function was gradually recovering.

Scr, BUN and UA are important indicators of chronic renal failure [17]. When kidney injury occurs, uric acid and creatinine cannot be excreted from the kidney, serum creatinine level increases, glomerular filtration rate decreases, and then blood urea nitrogen level increases rapidly [18]. In this study, Scr, BUN and UA levels were increased in the model group, while serum indexes were improved in the treatment group, indicating that the degree of renal damage in rats was significantly reduced. Shenkang Injection and its pharmacodynamic components showed good protective effects on renal function.

Epithelial-Mesenchymal-Transition(EMT) of renal tubule epithelial cells is one of the important mechanisms in the development of renal tubule interstitial fibrosis [19]. After fibrosis, the ability of renal tubular epithelial cells to adhere is lost, which is mainly marked by reduced or absent expression of E-cadherin [20]. The positive expression of α-SMA protein is a marker of myofibroblasts, and its increased expression level indicates that renal tubular epithelial cells are transformed into myofibroblasts, which have a strong ability to produce ECM and secrete a large amount of type I and type II collagen, which is deposited in the extracellular interstitium, leading to renal interstitial fibrosis and renal function decline [21]. In this study, compared with the normal control group, the expression of E-cadherin in the kidney of model group was significantly decreased, and the expression of α-SMA protein was increased, indicating the occurrence of renal fibrosis. Compared with the model group, the expression of E-cadherin in Shenkang injection group and mixed drug group increased, and the expression of α-SMA protein decreased, indicating that the degree of fibrosis in the drug treatment group was relieved.

Among the pharmacodynamic components of shenkang injection, HSYA comes from safflower, which can inhibit platelet aggregation and release induced by platelet-activating factors, and can competitively inhibit the binding of platelet-activating factors and platelet receptors. HSYA is an effective component of safflower yellow pigment in promoting blood circulation and removing blood stasis [22]. Tanshinol, derived from Salvia miltiorrhiza, can improve the damage degree of blood vessels and enhance their growth ability, and can effectively play an anti-inflammatory role [23]. It can also reduce the expression of inflammatory factor genes by targeting the inhibition of PI3K/AKT signaling pathway, so as to play an anti-fibrosis role [24]. Rheum emodin is derived from rhubarb, a traditional Chinese medicine. It has been reported that it plays an important role in the anti-renal fibrosis. After emodin treatment, proteins related to renal fibrosis are decreased, and expressions of LC3 and Beclin-1 are increased, suggesting that the anti-renal fibrosis effect of emodin is related to the increase of autophagy [25]. Astragaloside IV is derived from Astragaloside. Astragaloside IV can reduce the overexpression of fibrotic factors and reverse the Epithelial-mesenchymal transition (EMT) to delay the progression of renal fibrosis [26]. The four pharmacological components screened in this study are representative components of Shenkang Injection. Based on the orthogonal test, the optimal ratio was obtained, and the best anti-chronic renal failure effect was played with each other.

PI3K/AKT signaling pathway can induce renal hypoperfusion, accelerated extracellular matrix deposition and renal interstitial mononuclear infiltration by inducing vascular remodeling, activating enzyme response, changing hemodynamics and triggering inflammatory cascade reactions, leading to renal interstitial inflammatory injury and renal interstitial fibrosis [27]. In this study, the rat model of chronic renal failure was established to observe the mRNA and protein expression of PI3K/AKT signaling pathway and the pathological changes of rat kidney. The results of this study showed that compared with the control group, the mRNA expression levels and protein expression of target genes PI3K and AKT were increased in the model group, and the expression inhibition was obvious in all treatment groups, indicating that Shenkang injection and its components play the anti-fibrosis effect through this pathway.

The study also had its limitations. In this paper, adenine-induced renal failure rats were used as animal models. However, the main causes of chronic renal failure in clinical practice were glomerulonephritis, renal tubular disease, renal interstitial disease, diabetes, high blood pressure, immune diseases such as lupus and vasculitis, etc. Therefore, the model adopted in this study might not be clinically representative. The model used in this study belongs to clinically obstructive nephropathy, Shenkang injection treatment for these patients will had a good effect. At present, there had been many studies on Shenkang injection in the treatment of nephropathy, and the key pathways of research also varied. Some studies suggested that Wnt/β-Catenin Pathway was a critical pathway for treatment, and other studies suggested that JAK2/STAT3 or MAPK signaling pathways also played a key role [28–30]. After analysis, it was found that some of them were used to treat chronic kidney injury, some treat renal fibrosis and some focus on oxidative stress. Therefore, due to the inconsistency of research objects, the key pathways of attention were also correspondingly inconsistent. The above studies had achieved good results, laying a good foundation for the further development of Shenkang injection.

## Conclusions

In this study, the main pharmacodynamic substances and key pathways of Shenkang injection in the treatment of chronic renal failure were screened by network pharmacology technology. The degree of binding between pharmacodynamic substances and target proteins was verified by molecular docking technology. Meanwhile, the optimal ratio of pharmacodynamic components was optimized by orthogonal test. Target gene expression and signaling pathway expression. The results show that Shenkang injection has a good therapeutic effect on chronic renal failure, which may be achieved by pharmacodynamic substances such as HSYA, tanshinol, rheum emodin, Astragaloside IV, and the regulation of PI3K-Akt signaling pathway. The main innovation of this study is that the potential pharmacodynamic components have been discovered through scientific means, and the experimental verification has achieved good results, which may become a new drug for the treatment of renal failure, and provide novel reference ideas for the future research of related traditional Chinese medicine and the development of target drugs.

## Supporting information

S1 Table Identification of 90 chemical compounds of Shenkang injection by using UHPLC-Q-Orbitrap-MS/MS. (DOC)

S2 Table Pathological evaluation criteria. (DOC)

S3 Table The degree value of each component. (DOC)

S4 Table Molecular docking result. (DOC)

S1 Raw images (PDF)

S1 Graphical abstract (TIF)

## References

[1] Eyeni Sinomono DT, Loumingou R, Gassongo Koumou GC, Mahoungou GH, Mobengo JL. Chronic renal failure in the brazzaville university hospital center: Epidemiological, clinical and evolutionary aspects. Saudi J Kidney Dis Transpl. 2021 Sep-Oct;32(5):1450–1455. doi: 10.4103/1319-2442.344766 35532716

[2] Gao R, Yang B, Chen C, Chen F, Chen C, Zhao D, et al. Recognition of chronic renal failure based on Raman spectroscopy and convolutional neural network. Photodiagnosis Photodyn Ther. 2021 Jun;34:102313. doi: 10.1016/j.pdpdt.2021.102313 33915311

[3] Flagg AJ. Chronic Renal Therapy. Nurs Clin North Am. 2018 Dec;53(4):511–519. doi: 10.1016/j.cnur.2018.07.002 30388977

[4] Wang X, Wang ZY, Zheng JH, Li S. TCM network pharmacology: A new trend towards combining computational, experimental and clinical approaches. Chin J Nat Med. 2021 Jan;19(1):1–11. doi: 10.1016/S1875-5364(21)60001-8 33516447

[5] Xie M, Tao W, Wu F, Wu K, Huang X, Ling G, et al. Anti-hypertensive and cardioprotective activities of traditional Chinese medicine-derived polysaccharides: A review. Int J Biol Macromol. 2021 Aug 31;185:917–934. doi: 10.1016/j.ijbiomac.2021.07.008 34229020

[6] Wang Z, Zhang S, Zheng X, Zhang L. Efficacy and safety of colonic dialysis combined with traditional Chinese medicine retention enema in the treatment of chronic renal failure: A protocol for systematic review and meta-analysis. Medicine (Baltimore). 2021 Dec 17;100(50):e28082. doi: 10.1097/MD.0000000000028082 34918661 PMC8677955

[7] Luo LP, Suo P, Ren LL, Liu HJ, Zhang Y, Zhao YY. Shenkang Injection and Its Three Anthraquinones Ameliorates Renal Fibrosis by Simultaneous Targeting IƙB/NF-ƙB and Keap1/Nrf2 Signaling Pathways. Front Pharmacol. 2021 Dec 22;12:800522. 35002735 10.3389/fphar.2021.800522 PMC8729217

[8] Zou JJ, Zhou XT, Chen YK, Liu JL, Wang C, Ma YR, et al. A review on the efficacy and mechanism of action of Shenkang injection against chronic kidney disease. Biomed Pharmacother. 2020 Dec;132:110833. doi: 10.1016/j.biopha.2020.110833 33035831

[9] Xu T, Zuo L, Sun Z, et al. Chemical profiling and quantification of ShenKang injection, a systematic quality control strategy using ultra high performance liquid chromatography with Q Exactive hybrid quadrupole orbitrap high-resolution accurate mass spectrometry. J Sep Sci. 2017;40(24):4872–4879. doi: 10.1002/jssc.201700928 29106064

[10] Wang Y, Li M, Li C, Xu S, Wu J, Zhang G, et al. Efficacy and safety of Shenkang injection as adjuvant therapy in patients with diabetic nephropathy: A protocol for systematic review and meta-analysis. Medicine (Baltimore). 2020 Dec 24;99(52):e23821. doi: 10.1097/MD.0000000000023821 33350769 PMC7769367

[11] Xing RL, Meng DM, Ren W. Exploration on the Establishment of Anima Models for Gouty Nephropathy Complicated with Chronic Rena Failure. Zhongguo Zhong Xi Yi Jie He Za Zhi. 2011;31(10):1409–1413. 22097216

[12] Zhou Y, Jiang H, Huang X, et al. Indistinct assessment of the quality of traditional Chinese medicine in precision medicine exampling as safflower. J Pharm Biomed Anal. 2023;227:115277. doi: 10.1016/j.jpba.2023.115277 36736110

[13] Li Q, Qi L, Zhao K, Ke W, Li T, Xia L. Integrative quantitative and qualitative analysis for the quality evaluation and monitoring of Danshen medicines from different sources using HPLC-DAD and NIR combined with chemometrics. Front Plant Sci. 2022;13:932855. doi: 10.3389/fpls.2022.932855 36325569 PMC9618615

[14] Liang C, Yao Y, Ding H, Li X, Li Y, Cai T. Rapid classification and identification of chemical components of Astragali radix by UPLC-Q-TOF-MS. Phytochem Anal. 2022;33(6):943–960. doi: 10.1002/pca.3150 35726352

[15] Gourgiotis S, Germanos S, Dimopoulos N, Vougas V, Anastasiou T, Baratsis S. Renal injury: 5-year experience and literature review. Urol Int. 2006;77(2):97–103. doi: 10.1159/000093899 16888410

[16] Yee J, Parasuraman R, Narins RG. Selective review of key perioperative renal-electrolyte disturbances in chronic renal failure patients. Chest. 1999 May;115(5 Suppl):149S–157S. doi: 10.1378/chest.115.suppl_2.149s 10331349

[17] Zou C, Wu YC, Lin QZ. Effects of Chinese herbal enema therapy combined basic treatment on BUN, SCr, UA, and IS in chronic renal failure patients. Zhongguo Zhong Xi Yi Jie He Za Zhi. 2012 Sep;32(9):1192–5. 23185756

[18] Liu Y, Hu Q, Shen P, Tang L, Yuan G, Zhou Y, et al. Clinical and pathological analysis of IgA nephropathy with chronic renal failure. Ren Fail. 2016 Oct;38(9):1347–1352. doi: 10.1080/0886022X.2016.1214051 27558026

[19] Xu Z, Jia K, Wang H, Gao F, Zhao S, Li F, et al. METTL14-regulated PI3K/Akt signaling pathway via PTEN affects HDAC5-mediated epithelial-mesenchymal transition of renal tubular cells in diabetic kidney disease. Cell Death Dis. 2021 Jan 4;12(1):32. doi: 10.1038/s41419-020-03312-0 33414476 PMC7791055

[20] Sun L, Dong H, Zhang W, Wang N, Ni N, Bai X, et al. Lipid Peroxidation, GSH Depletion, and SLC7A11 Inhibition Are Common Causes of EMT and Ferroptosis in A549 Cells, but Different in Specific Mechanisms. DNA Cell Biol. 2021 Feb;40(2):172–183. doi: 10.1089/dna.2020.5730 33351681

[21] Wang YY, Jiang H, Pan J, Huang XR, Wang YC, Huang HF, et al. Macrophage-to-Myofibroblast Transition Contributes to Interstitial Fibrosis in Chronic Renal Allograft Injury. J Am Soc Nephrol. 2017 Jul;28(7):2053–2067. doi: 10.1681/ASN.2016050573 28209809 PMC5491278

[22] Fu S, Zhou Q, Gao Y, Yang Y, Chen H, Yuan L, et al. Antioxidant and Anti-Inflammatory Properties of Hydroxyl Safflower Yellow a in Diabetic Nephropathy: A Meta-Analysis of Randomized Controlled Trials. Front Pharmacol. 2022 Aug 11;13:929169. doi: 10.3389/fphar.2022.929169 36034830 PMC9404325

[23] Li M, Wang F, Huang Y, Du F, Zhong C, Olaleye OE, et al. Systemic exposure to and disposition of catechols derived from Salvia miltiorrhiza roots (Danshen) after intravenous dosing DanHong injection in human subjects, rats, and dogs. Drug Metab Dispos. 2015 May;43(5):679–90. doi: 10.1124/dmd.114.061473 25670806

[24] Zhou J, Jiang YY, Chen H, Wu YC, Zhang L. Tanshinone I attenuates the malignant biological properties of ovarian cancer by inducing apoptosis and autophagy via the inactivation of PI3 K/AKT/mTOR pathway. Cell Prolif. 2020 Feb;53(2):e12739. 10.1111/cpr.12739 PMC7046305 31820522

[25] Wang XT, Sun XJ, Li C, Liu Y, Zhang L, Li YD, et al. Establishing a Cell-Based High-Content Screening Assay for TCM Compounds with Anti-Renal Fibrosis Effects. Evid Based Complement Alternat Med. 2018 Jun 28;2018:7942614. doi: 10.1155/2018/7942614 30050593 PMC6046160

[26] Qian W, Cai X, Qian Q, Zhang W, Wang D. Astragaloside IV modulates TGF-β1-dependent epithelial-mesenchymal transition in bleomycin-induced pulmonary fibrosis. J Cell Mol Med. 2018 Sep;22(9):4354–4365. 29971947 10.1111/jcmm.13725 PMC6111865

[27] Tu H, Ma D, Luo Y, Tang S, Li Y, Chen G, et al. Quercetin alleviates chronic renal failure by targeting the PI3k/Akt pathway. Bioengineered. 2021 Dec;12(1):6538–6558. doi: 10.1080/21655979.2021.1973877 34528858 PMC8806539

[28] Wei HT, Xu Y, Tan XY, Jing HY, Ma YR. ShenKang Injection Attenuates Renal Fibrosis by Inhibiting EMT and Regulating the Wnt/β-Catenin Pathway. Evid Based Complement Alternat Med. 2022;2022:9705948. Published 2022 Jun 28. doi: 10.1155/2022/9705948 35800011 PMC9256403

[29] Qin T, Wu Y, Liu T, Wu L. Effect of Shenkang on renal fibrosis and activation of renal interstitial fibroblasts through the JAK2/STAT3 pathway. BMC Complement Med Ther. 2021;21(1):12. Published 2021 Jan 6. doi: 10.1186/s12906-020-03180-3 33407391 PMC7789243

[30] Liu M, Park J, Wu X, et al. Shen-Kang protects 5/6 nephrectomized rats against renal injury by reducing oxidative stress through the MAPK signaling pathways. Int J Mol Med. 2015;36(4):975–984. doi: 10.3892/ijmm.2015.2328 26310779 PMC4564094

