# Supplementary material for: Study on the mechanism of Shenkang injection in the treatment of chronic renal failure based on the strategy of "Network pharmacology—Molecular docking—Key target validation"
Source: PLoS One. 2023 Oct 5;18(10):e0291621. doi: 10.1371/journal.pone.0291621 (PMC10553805; doi:10.1371/journal.pone.0291621)
Supplement: S1 Table — (DOC) [file pone.0291621.s001.doc]

Table S1 Identification of 90 chemical compounds of Shenkang Injection by using UHPLC-Q-Orbitrap-MS/MS

| No. | Time | Formula | compounds | Ion  mode | ES/expected  (m/z) | ES / measured  (m/z) | Delta  (ppm) | HPLC-ESI-MS/MS (m/z) |
| --- | --- | --- | --- | --- | --- | --- | --- | --- |
|  | 1.26 | C_13_H_16_O_10_ | Gallicacid-4-*O-β*-D-glucoside | ESI^-^ | 331.06706 | 331.06699 | -0.241 | **331.06744**; 169.01350(C_7_H_5_O_5_);  125.02332(C_6_H_5_O_3_) |
|  | 1.41 | C_7_H_6_O_5_ | gallic acid ^a^ | ESI^-^ | 169.01424 | 169.01324 | -5.955 | **169.01344**;125.02329(C_6_H_5_O_3_); 97.02820(C_5_H_5_O_2_) |
|  | 1.41 | C_6_H_6_O_3_ | 5-hydroxymethylfurfural | ESI^-^ | 125.02441 | 125.02318 | -9.897 | **125.02335** |
|  | 1.49 | C_19_H_26_O_15_ | 4'-*O*-galloylsucrose/1'-*O*-galloylsucrose | ESI^-^ | 493.11989 | 493.11932 | -1.162 | **493.12054**;331.06744(C_13_H_15_O_10_); 271.04626(C_11_H_11_O_8_); 169.01346(C_7_H_5_O_5_) |
|  | 1.82 | C_13_H_16_O_10_ | Gallic  acid-3-*O-β*-D-glucoside | ESI^-^ | 331.06706 | 331.06717 | 0.303 | 169.01344(C_7_H_5_O_5_); 125.02328(C_6_H_5_O_3_) |
|  | 1.90 | C_10_H_12_O_7_ | 1-*O*-galloylycerol | ESI^-^ | 243.05102 | 243.05080 | -0.765 | **243.05092**; 169.01347(C_7_H_5_O_5_) |
|  | 1.99 | C_8_H_8_O_4_ | vanillic acid/its isomer | ESI^-^ | 167.03498 | 167.03391 | -6.418 | **167.03416**;149.02364(C_8_H_5_O_3_); 123.04402(C_7_H_7_O_2_) |
|  | 2.04 | C_9_H_10_O_5_ | tanshinol ^a^ | ESI^-^ | 197.04554 | 197.04469 | -4.348 | **197.04504**;179.03426(C_9_H_7_O_4_); 135.04411(C_8_H_7_O_2_);123.04401(C_7_H_7_O_2_); 72.9918(C_2_HO_3_) |
|  | 2.17 | C_8_H_8_O_4_ | vanillic acid/its isomer | ESI^-^ | 167.03498 | 167.03401 | -5.819 | **167.03416**;149.02364(C_8_H_5_O_3_); 123.04406(C_7_H_7_O_2_); |
|  | 2.38 | C_7_H_6_O_4_ | protocatechuic acid ^a^ | ESI^-^ | 153.01933 | 153.01834 | -6.483 | **153.01842**; 109.02831(C_6_H_5_O_2_) |
|  | 2.88 | C_16_H_18_O_9_ | chlorogenic acid-isomer | ESI^-^ | 353.08780 | 353.08789 | 0.240 | 191.05560(C_7_H_11_O_6_); 179.03418(C_9_H_7_O_4_); 135.04405(C_8_H_7_O_2_); |
|  | 2.96 | C_8_H_8_O_5_ | methyl gallate/its isomer | ESI^-^ | 183.02989 | 183.02913 | -4.188 | **183.02930**;168.00565(C_7_H_4_O_5_); 139.03906(C_7_H_7_O_3_); 124.01544(C_6_H_4_O_3_) |
|  | 3.01 | C_8_H_8_O_3_ | vanillin | ESI^-^ | 151.04006 | 151.03905 | -6.736 | **151.03914**;123.04398(C_7_H_7_O_2_);  107.04900(C_7_H_7_O) |
|  | 3.16 | C_8_H_8_O_5_ | methyl gallate/its isomer | ESI^-^ | 183.02989 | 183.02911 | -4.298 | **183.02930**;168.00565(C_7_H_4_O_5_); 139.03906(C_7_H_7_O_3_); 124.01544(C_6_H_4_O_3_) |
|  | 3.23 | C_15_H_14_O_6_ | catechin -isomer | ESI^+^ | 291.08631 | 291.08582 | -1.699 | **291.08615**;139.03879(C_7_H_7_O_3_); 123.04400(C_7_H_7_O_2_) |
|  | 3.38 | C_7_H_6_O_3_ | protocatechu aldehyde ^a^ | ESI^-^ | 137.02441/139.03897 | 137.02313/139.03888 | -9.395/-0.652 | **137.02341**;93.03332(C_6_H_5_O)/**139.03877**; 111.04411(C_6_H_7_O_2_); 93.03378(C_6_H_5_O_1_) |
|  | 3.46 | C_27_H_32_O_16_ | hydroxysafflor yellow A-isomer | ESI^-^ | 611.16175 | 611.16113 | -1.027 | **611.16187**;521.12994(C_24_H_25_O_13_); 491.12009(C_23_H_23_O_12_); 473.10962(C_23_H_21_O_11_) |
|  | 3.55 | C_33_H_40_O_22_ | 6-hydroxykaempferol-3, 6, 7-tri-O-β-D-glucoside | ESI^-^/  ESI^+^ | 787.19384/789.20839 | 787.19257/789.20679 | -1.621/-2.039 | **787.19324**;625.14197(C_27_H_29_O_17_); 463.08905(C_21_H_19_O_12_), 301.03552(C_15_H_9_O_7_)  /465.10184(C_21_H_21_O_12_);303.04926(C_15_H_11_O_7_) |
|  | 3.55 | C_20_H_20_O_14_ | 2,6-di-galloyglucose | ESI^-^ | 483.0780 | 483.07788 | -0.307 | 169.01355(C_7_H_5_O_5_) |
|  | 3.55 | C_15_H_14_O_6_ | catechin ^a^ | ESI^+^ | 291.08631 | 291.08609 | -0.772 | **291.08615**;139.03879(C_7_H_7_O_3_); 123.04399(C_7_H_7_O_2_) |
|  | 3.68 | C_16_H_18_O_9_ | chlorogenic acid ^a^ | ESI^-^ | 353.08780 | 353.08759 | -0.610 | 191.05553(C_7_H_11_O_6_) |
|  | 3.75 | C_27_H_32_O_16_ | hydroxysafflor yellow A ^a^ | ESI^-^/ESI^+^ | 611.16175/613.17631 | 611.16150/613.17542 | -0.831/-1.258 | **611.16223**;491.11996(C_23_H_23_O_12_);473.10992(C_23_H_21_O_11_);403.10388(C_20_H_19_O_9_);325.07220(C_18_H_13_O_6_)/451.12274(C_21_H_23_O_11_);331.08075(C_17_H_15_O_7_);313.07187(C_17_H_13_O_6_); 211.02330( C_9_H_7_O_6_); 147.04373 ( C_9_H_7_O_2_); |
|  | 3.77 | C_20_H_20_O_14_ | gallic acid-4-*O-β*-D-(6'-*O*-galloyl)-glucoside | ESI^-^ | 483.07802 | 483.07773 | -0.618 | **483.07803**;313.05688(C_13_H_13_O_9_); 169.01347(C_7_H_5_O_5_) |
|  | 3.81 | C_27_H_30_O_17_ | 6-hydroxykaempferol-3, 6-di-O-β-D-glucoside | ESI^-^/ESI^+^ | 625.14102/627.15557 | 625.14081/627.15454 | -1.220/-1.651 | **625.14111**;463.08835(C_21_H_19_O_12_); 462.08115(C_21_H_18_O_12_); 301.03561(C_15_H_9_O_7_); 299.02008(C_15_H_7_O_7_)  /465.10156(C_21_H_21_O_12_);303.04932(C_15_H_11_O_7_) |
|  | 3.82 | C_10_H_10_O_4_ | ferulic acid-isomer | ESI^-^/ESI^+^ | 193.05063 | 193.04990 | -3.792 | 165.01854(C_8_H_5_O_4_); 149.05988(C_9_H_9_O_2_) |
|  | 3.85 | C_20_H_20_O_14_ | gallic acid-3-*O-β*-D-(6'-*O*-galloyl)-glucoside | ESI^-^ | 483.07802 | 483.07788 | -0.307 | **483.07803**;313.05688(C_13_H_13_O_9_); 169.01347(C_7_H_5_O_5_) |
|  | 3.87 | C_16_H_18_O_9_ | chlorogenic acid-isomer | ESI^-^ | 353.08780 | 353.08786 | 0.155 | 191.05557(C_7_H_11_O_6_);179.03429(C_9_H_7_O_4_); 173.04483(C_7_H_9_O_5_); 135.04414(C_8_H_7_O_2_) |
|  | 3.99 | C_8_H_8_O_3_ | vanillin-isomer | ESI^-^ | 151.04006 | 151.03902 | -6.935 | **151.03920**;123.04437(C_7_H_7_O_2_);108.04442  (C_3_H_8_O_4_) |
|  | 4.03 | C_9_H_8_O_4_ | caffeic acid ^a^ | ESI^-^/ESI^+^ | 179.03498/181.04953 | 179.03419/181.04951 | -4.424/-0.140 | **179.03441**;135.04414(C_8_H_7_O_2_)  /163.03876(C_9_H_7_O_3_) |
|  | 4.17 | C_7_H_6_O_4_ | protocatechuic acid-isomer | ESI^-^ | 153.01933 | 153.01830 | -6.744 | **153.01842**; 109.02830(C_6_H_5_O_2_) |
|  | 4.48 | C_27_H_32_O_16_ | hydroxysafflor yellow A-isomer | ESI^-^ | 611.16175 | 611.16150 | -0.422 | **611.16241**;521.13025(C_24_H_25_O_13_); 449.10962(C_21_H_21_O_11_); 287.05637(C_15_H_11_O_6_) |
|  | 4.52 | C_27_H_30_O_17_ | 6-hydroxykaempferol-6, 7-di-O-β-D-glucoside | ESI^-^/ESI^+^ | 625.14102/627.15557 | 625.14038/627.15466 | -1.028/-1.460 | **625.14105**;463.08844(C_21_H_19_O_12_); 416.11191(C_21_H_20_O_9_); 301.03552(C_15_H_9_O_7_); 253.05066(C_15_H_9_O_4_)  /465.10156(C_21_H_21_O_12_);303.04932(C_15_H_11_O_7_) |
|  | 4.55 | C_7_H_6_O_4_ | protocatechuic acid-isomer | ESI^-^ | 153.01933 | 153.01825 | -7.017 | **153.01842**;135.00771(C_6_H_5_O_2_); 109.02831(C_7_H_3_O_3_) |
|  | 4.68 | C_22_H_20_O_11_ | carboxyl-chrysophanol-O-glucose/its isomer | ESI^-^ | 459.09328 | 459.09348 | 0.426 | 415.10385(C_21_H_19_O_9_); 253.05060(C_15_H_9_O_4_) |
|  | 5.01 | C_8_H_8_O_3_ | p-anisic acid /its isomer | ESI^-^ | 151.04006 | 151.03891 | -7.663 | **151.03922**;136.01552(C_7_H_4_O_3_); 107.04906(C_7_H_7_O) |
|  | 5.03 | C_21_H_20_O_11_ | kaempferol-3-O-β-D-glucoside/its isomer | ESI^-^ | 447.09328 | 447.09305 | -0.525 | **447.09341**; 285.04065(C_15_H_9_O_6_) |
|  | 5.14 | C_21_H_20_O_10_ | aloe-emodin-1-*O-β*-D- glucoside | ESI^-^ | 431.09837 | 431.09827 | -0.232 | 293.04581(C_17_H_9_O_5_); 269.04575(C_15_H_9_O_5_) |
|  | 5.21 | C_27_H_22_O_12_ | lithospermic acid/its isomer | ESI^-^ | 537.10384 | 537.10382 | -0.054 | 295.06137(C_17_H_11_O_5_); 185.02376(C_11_H_5_O_3_); 109.02830(C_6_H_5_O_2_) |
|  | 5.21 | C_21_H_18_O_11_ | rhein-8-O-β-D-glucoside | ESI^-^ | 445.07763 | 445.07767 | 0.080 | 283.02499(C_15_H_7_O_6_); 239.03482(C_14_H_7_O_4_) |
|  | 5.29 | C_10_H_10_O_4_ | ferulic acid ^a^ | ESI^-^/ESI^+^ | 193.05063/195.06518 | 193.04976/195.06490 | -4.517/-1.463 | **193.05008**;178.02644(C_9_H_6_O_4_);149.05984(C_9_H_9_O_2_);134.03630(C_8_H_6_O_2_)  /177.05434(C_10_H_9_O_3_);145.02817(C_9_H_5_O_2_); 89.06004(C_4_H_9_O_2_) |
|  | 5.29 | C_23_H_26_O_11_ | lindleyin/isolindleyin | ESI^-^/ESI^+^ | 477.14023/479.15478 | 477.14001/479.15363 | -0.471/-2.417 | **477.14206**; 313.05701(C_13_H_13_O_9_)  169.01346(C_7_H_5_O_5_)  /255.04916(C_11_H_11_O_7_); 153.01799(C_7_H_5_O_4_) |
|  | 5.34 | C_27_H_22_O_12_ | lithospermic acid/its isomer | ESI^-^ | 537.10384 | 537.10364 | -0.390 | 295.06134(C_17_H_11_O_5_); 185.02377(C_11_H_5_O_3_); 109.02830(C_6_H_5_O_2_) |
|  | 5.67 | C_22_H_20_O_11_ | carboxyl-chrysophanol-O-glucose/its isomer | ESI^-^ | 459.09328 | 459.09311 | -0.380 | 253.05063(C_15_H_9_O_4_); 173.06004(C_11_H_9_O_2_) |
|  | 5.82 | C_21_H_20_O_11_ | kaempferol-3-O-β-D-glucoside/its isomer | ESI^-^ | 447.09328 | 447.09348 | 0.437 | **447.08838**; 284.03287(C_15_H_8_O_6_) |
|  | 5.82 | C_36_H_30_O_16_ | salvianolic acid B-isomer | ESI^-^ | 717.14610 | 717.14594 | -0.234 | 519.09375(C_27_H_19_O_11_);339.05112(C_18_H_11_O_7_); 321.04074(C_18_H_9_O_6_); 295.06128(C_17_H_11_O_5_) |
|  | 6.02 | C_18_H_16_O_8_ | rosmarinic acid ^a^ | ESI^-^ | 359.07724 | 359.07730 | 0.165 | **359.07822**;197.04506(C_9_H_9_O_5_); 179.03436(C_9_H_7_O_4_); 161.02357(C_9_H_5_O_3_) |
|  | 6.09 | C_21_H_20_O_11_ | kaempferol-3-O-β-D-glucoside/its isomer | ESI^-^ | 447.09328 | 447.09332 | 0.079 | **447.09290**; 284.03287(C_15_H_8_O_6_) |
|  | 6.13 | C_9_H_16_O_4_ | azelaic acid | ESI^-^ | 187.09758 | 187.09679 | -4.234 | **187.09698**; 125.09610(C_8_H_13_O) |
|  | 6.17 | C_30_H_30_O_14_ | isosafflomin C | ESI^-^ | 613.15627 | 613.15582 | -0.748 | **613.15692**;595.14612(C_30_H_27_O_13_);551.15778(C_29_H_27_O_11_);425.11072(C_19_H_21_O_11_);  361.10852(C_22_H_17_O_5_); 287.05597(C_15_H_11_O_6_) |
|  | 6.21 | C_21_H_20_O_10_ | aloe-emodin-8-*O-β*-D- glucoside | ESI^-^ | 431.09837 | 431.09821 | -0.371 | **431.09857**;269.04568(C_15_H_9_O_5_); 240.04254(C_14_H_8_O_4_) |
|  | 6.25 | C_7_H_6_O_3_ | protocatechu aldehyde -isomer | ESI^-^ | 137.02441 | 137.02325 | -8.519 | **137.02341**; 93.03333(C_6_H_5_O) |
|  | 6.30 | C_30_H_30_O_14_ | safflomin C | ESI^-^ | 613.15627 | 613.15588 | -0.650 | **613.15674**;595.14587(C_30_H_27_O_13_);551.15686(C_29_H_27_O_1_); 425.10901(C_19_H_21_O_11_);  361.10864(C_22_H_17_O_5_); 287.05569(C_15_H_11_O_6_) |
|  | 6.35 | C_36_H_30_O_16_ | salvianolic acid B ^a^ | ESI^-^ | 717.14610 | 717.14575 | -0.499 | 519.09369(C_27_H_19_O_11_);339.05127(C_18_H_11_O_7_); 321.04083(C_18_H_9_O_6_); 295.06140(C_17_H_11_O_5_) |
|  | 6.58 | C_27_H_30_O_15_ | safflor yellow A | ESI^-^ | 593.15119 | 593.15112 | -0.124 | **593.15186**; 269.04584(C_15_H_9_O_5_) |
|  | 6.63 | C_22_H_20_O_11_ | carboxyl-chrysophanol-O-glucose/its isomer | ESI^-^ | 459.09328 | 459.09348 | 0.426 | 266.05850(C_16_H_10_O_4_); 253.05061(C_15_H_9_O_4_) |
|  | 6.68 | C_22_H_22_O_9_ | formononetin | ESI^+^ | 431.13365 | 431.13287 | -1.829 | 269.08032(C_16_H_13_O_4_) |
|  | 7.14 | C8H8O4 | p-anisic acid /its isomer | ESI^-^ | 151.04006 | 151.03896 | -7.332 | 151.03917;136.01563(C7H4O3); 107.04905(C7H7O) |
|  | 7.17 | C_23_H_26_O_10_ | 9,10-dimethoxyisoflavone-3-O-glucose | ESI^+^ | 463.15987 | 463.15845 | -3.073 | 301.10635 (C_17_H_17_O_5_); 167.07004 (C_9_H_11_O_3_) |
|  | 7.44 | C_26_H_20_O_10_ | salvianolic acid C/its isomer | ESI^-^ | 491.09837 | 491.09848 | 0.224 | **491.09918**;311.05646(C_17_H_11_O_6_); 293.04593(C_17_H_9_O_5_); 135.04413(C_8_H_7_O_2_) |
|  | 7.46 | C_23_H_28_O_10_ | isomucronulatol-7-O- glucose | ESI^+^ | 465.17552 | 465.17447 | -2.265 | 303.19489(C_19_H_27_O_3_); 167.07004 (C_9_H_11_O_3_); 123.04401(C_7_H_7_O_2_) |
|  | 7.62 | C_21_H_20_O_9_ | chrysophanol-1-*O-β*-D- glucoside | ESI^-^ | 415.10345 | 415.10336 | -0.230 | 253.05067(C_15_H_9_O_4_) |
|  | 7.79 | C_21_H_20_O_10_ | emodin-1-*O-β*-D- glucoside | ESI^-^/ESI^+^ | 431.09837/433.11292 | 431.09802/433.11157 | -0.812/-3.124 | **431.09882**;269.04578(C_15_H_9_O_5_)  /313.07004(C_17_H_13_O_6_); 271.05966(C_15_H_11_O_5_) |
|  | 7.83 | C_16_H_10_O_7_ | laccic acid D | ESI^-^ | 313.03537 | 313.03519 | -0.594 | 269.04581(C_15_H_9_O_5_) |
|  | 7.94 | C_21_H_20_O_9_ | chrysophanol-8-*O-β*-D- glucoside | ESI^-^ | 415.10345 | 415.10342 | -0.085 | 253.05067(C_15_H_9_O_4_) |
|  | 8.13 | C_26_H_20_O_10_ | salvianolic acid C/its isomer | ESI^-^ | 491.09837 | 491.09827 | -0.204 | 293.04590(C_17_H_9_O_5_) |
|  | 8.27 | C_15_H_12_O_5_ | naringenin ^a^ | ESI^-^/ESI^+^ | 271.06119/273.07575 | 271.06110/273.07507 | -0.357/-2.490 | **271.06152**;151.00279(C_7_H_3_O_4_);119.04920(C_8_H_7_O)/**273.18469**;231.10152(C_14_H_15_O_3_) |
|  | 8.83 | C_15_H_10_O_6_ | omg-hydroxy-emodin/its isomer | ESI^-^ | 285.04046 | 285.04047 | 0.031 | **285.04047**;257.04523(C_14_H_9_O_5_); 241.05020(C_14_H_9_O_4_) |
|  | 9.03 | C_16_H_12_O_5_ | calycosin | ESI^+^ | 285.07575 | 285.07507 | -2.385 | **285.07520** |
|  | 9.04 | C_22_H_22_O_10_ | physcion-8-*O-β*-D-glucoside | ESI^-^ | 445.11402 | 445.11398 | -0.090 | **445.11417**;283.06143(C_16_H_11_O_5_); 240.04259(C_14_H_8_O_4_); |
|  | 9.07 | C_21_H_20_O_10_ | emodin-8-*O-β*-D-glucoside | ESI^-^ | 431.09837 | 431.09802 | -0.812 | **431.09824**; 269.04587(C_15_H_9_O_5_) |
|  | 9.15 | C_19_H_20_O_4_ | tanshinone IIB/its isomer | ESI^+^ | 313.14343 | 313.14279 | -2.062 | **313.14282**; 269.15314(C_18_H_21_O_2_) |
|  | 9.36 | C_15_H_10_O_6_ | omg-hydroxy-emodin/its isomer | ESI^-^ | 285.04046 | 285.04047 | 0.031 | **285.04077**; 241.05049(C_14_H_9_O_4_) |
|  | 9.38 | C_23_H_22_O_11_ | aloe-emodin-8-O-(6-O-acetyl)-glucoside | ESI^-^ | 473.10893 | 473.10873 | -0.433 | **473.10968**;311.05710(C_17_H_11_O_6_); 269.04584(C_15_H_9_O_5_) |
|  | 9.67 | C_19_H_20_O_3_ | cryptotanshinone /its isomer | ESI^+^ | 297.14852 | 297.14804 | -1.619 | 253.15822(C_18_H_21_O); 238.13481(C_17_H_18_O) |
|  | 9.80 | C_19_H_16_O_4_ | tanshinaldehyde/ its isomer | ESI^+^ | 309.11213 | 309.11148 | -2.121 | **309.11111**; 265.12183(C_18_H_17_O_2_) |
|  | 9.91 | C_22_H_22_O_10_ | physcion-1-*O-β*-D-glucoside | ESI^-^ | 445.11402 | 445.11389 | -0.090 | **445.11459**; 283.06140(C_16_H_11_O_5_) |
|  | 10.13 | C_19_H_20_O_4_ | tanshinone IIB/its isomer | ESI^+^ | 313.14343 | 313.14258 | -2.732 | **313.14243**;295.13229(C_19_H_19_O_3_); 251.14247(C_18_H_19_O) |
|  | 10.26 | C_15_H_10_O_4_ | chrysophanol -isomer | ESI^-^ | 253.05063 | 253.05028 | -1.391 | **253.05063** |
|  | 10.26 | C_16_H_10_O_6_ | 6-methyl-rhein | ESI^-^ | 297.04046 | 297.04028 | -0.610 | **297.04074**;253.05061(C15H9O4); 225.05470(C14H9O3) |
|  | 10.30 | C_12_H_14_O_4_ | ethyl ferulate ^a^ | ESI^-^/ESI^+^ | 221.08193/223.09648 | 221.08136/223.09610 | -2.588/-1.728 | **221.08144**;177.09128(C_11_H_13_O_2_); 149.09608(C_10_H_13_O);134.03635(C_8_H_6_O_2_); 121.02837(C_7_H_5_O_2_);71.04897(C_4_H_7_O)  /149.02316(C_8_H_5_O_3_) |
|  | 10.52 | C_15_H_10_O_5_ | rheum emodin^a^ | ESI^-^/ESI^+^ | 269.04554/271.06009 | 269.04541/271.05966 | -0.396/-1.623 | **269.04578**;240.04272(C_14_H_8_O_4_); 225.05540(C_14_H_9_O_3_)/**271.05966**; 253.04913(C_15_H_9_O_4_) |
|  | 10.59 | C_41_H_68_O_14_ | astragaloside IV ^a^ | ESI^+^ | 785.46818 | 785.46552 | -3.390 | 473.36108(C_30_H_49_O_4_); 455.35211(C_30_H_47_O_3_); 437.34052(C_30_H_45_O_2_); 143.10654(C_8_H_15_O_2_) |
|  | 11.00 | C_15_H_8_O_6_ | rhein ^a^ | ESI^-^ | 283.02481 | 283.02460 | -0.746 | **283.02512**;257.04565(C_14_H_9_O_5_); 239.03484(C_14_H_7_O_4_) |
|  | 11.90 | C_19_H_16_O_4_ | tanshinaldehyde/ its isomer | ESI^+^ | 309.11213 | 309.11148 | -2.121 | **309.11081**; 265.12183(C_18_H_17_O_2_) |
|  | 12.13 | C_19_H_20_O_3_ | cryptotanshinone /its isomer | ESI^+^ | 297.14852 | 297.14789 | -2.124 | 253.15819(C_18_H_21_O);238.13481(C_17_H_18_O) |
|  | 12.13 | C_21_H_20_O_4_ | danshexinkum D | ESI^+^ | 337.14343 | 337.14020 | -9.597 | **337.14029** |
|  | 12.29 | C_19_H_22_O_4_ | tanshinone V | ESI^+^ | 315.15908 | 315.15826 | -2.620 | **315.15906**;297.14807(C_19_H_21_O_3_); 253.15822(C_18_H_21_O) |
|  | 12.30 | C_15_H_10_O_5_ | emodin ^a^ | ESI^-^ | 269.04554 | 269.04541 | -0.508 | **269.04554**;241.05089(C_14_H_9_O_4_);  225.05551(C_14_H_9_O_3_); |
|  | 13.20 | C_15_H_10_O_4_ | chrysophanol ^a^ | ESI^-^ | 253.05063 | 253.05028 | -1.391 | **253.05118**; 225.05591(C_14_H_9_O_3_) |
| 1. **^a^** | 13.58 | C_16_H_12_O_5_ | physcion ^a^ | ESI^-^/ESI^+^ | 283.06119/285.07575 | 283.06168/285.07547 | 1.707/-0.982 | **283.06192**; 240.04346(C_14_H_8_O_4_)  /**285.07532**; 270.05179(C_15_H_10_O_5_) |
