# Supplementary material for: Study on the mechanism of Shenkang injection in the treatment of chronic renal failure based on the strategy of "Network pharmacology—Molecular docking—Key target validation"
Source: PLoS One. 2023 Oct 5;18(10):e0291621. doi: 10.1371/journal.pone.0291621 (PMC10553805; doi:10.1371/journal.pone.0291621)
Supplement: S2 Table — (DOC) [file pone.0291621.s002.doc]

Table.S2 Pathological evaluation criteria

| Level (number) | Type | Description |
| --- | --- | --- |
| 0 | Within normal limits | Under the study conditions, the tissues were considered normal, taking into account factors such as age, sex and strain of the animals. Changes that occur under other conditions can be considered anomalies. |
| 1 | Very slight | The variation is just beyond the normal range |
| 2 | Slight | Lesions can be observed, but not serious |
| 3 | Intermediate | The lesions are obvious and probably more severe |
| 4 | Seriousness | The lesion is very severe (the lesion has occupied the entire tissue and organ) |
