# Supplementary material for: Study on the mechanism of Shenkang injection in the treatment of chronic renal failure based on the strategy of "Network pharmacology—Molecular docking—Key target validation"
Source: PLoS One. 2023 Oct 5;18(10):e0291621. doi: 10.1371/journal.pone.0291621 (PMC10553805; doi:10.1371/journal.pone.0291621)
Supplement: S3 Table — (DOC) [file pone.0291621.s003.doc]

| No | name | Degree | No | name | Degree |
| --- | --- | --- | --- | --- | --- |
| 1 | Hydroxysafflor yellow A | 80 | 11 | Calycosin | 30 |
| 2 | Rheum emodin | 47 | 12 | Jaranol | 30 |
| 3 | Tanshinol | 33 | 13 | Isoflavanone | 29 |
| 4 | Astragaloside IV | 33 | 14 | Formononetin | 29 |
| 5 | Flavoxanthin | 33 | 15 | MOL002260 | 29 |
| 6 | lupeol-palmitate | 32 | 16 | Toralactone | 29 |
| 7 | Phytoene | 31 | 17 | (-)-catechin | 28 |
| 8 | Baicalein | 31 | 18 | Kaempferol | 28 |
| 9 | Bifendate | 30 | 19 | Miltirone | 26 |
| 10 | Beta-carotene | 30 | 20 | Sennoside D_qt | 25 |

Table S3 The degree value of each component
