# Supplementary material for: Study on the mechanism of Shenkang injection in the treatment of chronic renal failure based on the strategy of "Network pharmacology—Molecular docking—Key target validation"
Source: PLoS One. 2023 Oct 5;18(10):e0291621. doi: 10.1371/journal.pone.0291621 (PMC10553805; doi:10.1371/journal.pone.0291621)
Supplement: S4 Table — (DOC) [file pone.0291621.s004.doc]

Table S4 Molecular docking result

| Protein/Compounds | HSYA | Tanshinol | Rheum emodin | Astragaloside IV | The optimal ligand |
| --- | --- | --- | --- | --- | --- |
| PI3K | -6.16 kcal/mol | -3.5 kcal/mol | -5.8 kcal/mol | -6.6 kcal/mol | -5.5 kcal/mol |
| Akt | -4.41 kcal/mol | -6.5 kcal/mol | -6.32 kcal/mol | -2.37 kcal/mol | -5.9 kcal/mol |
