# Supplementary figures and images for: Study on the mechanism of Shenkang injection in the treatment of chronic renal failure based on the strategy of "Network pharmacology—Molecular docking—Key target validation"
Source: PLoS One. 2023 Oct 5;18(10):e0291621. doi: 10.1371/journal.pone.0291621 (PMC10553805; doi:10.1371/journal.pone.0291621)

# p-PI3K

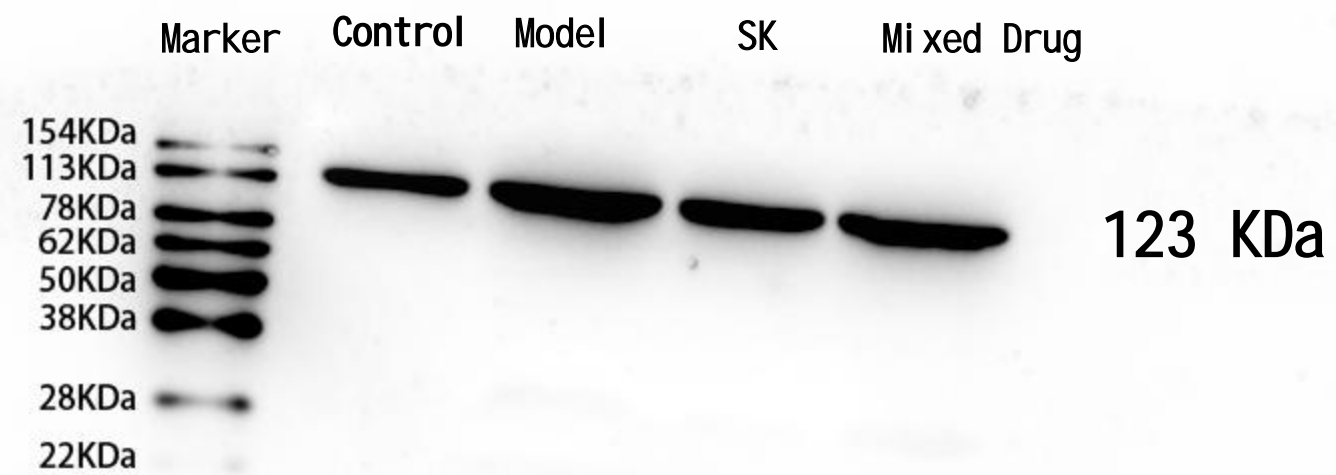

# PI3K

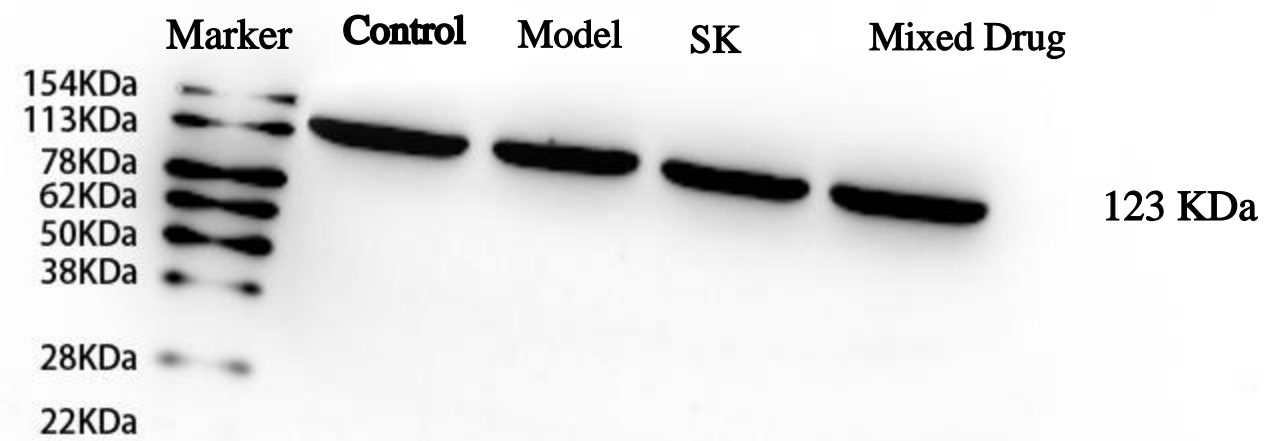

# p-AKT

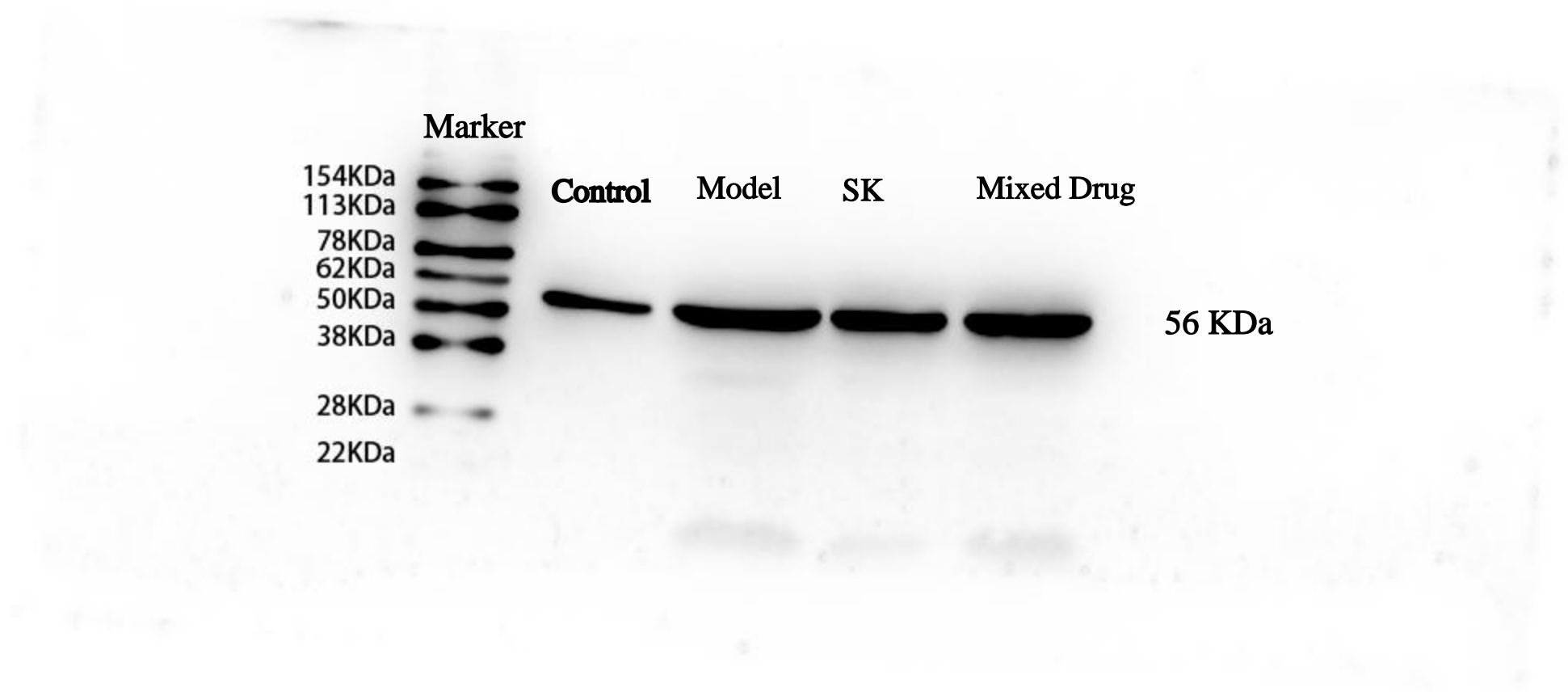

# AKT

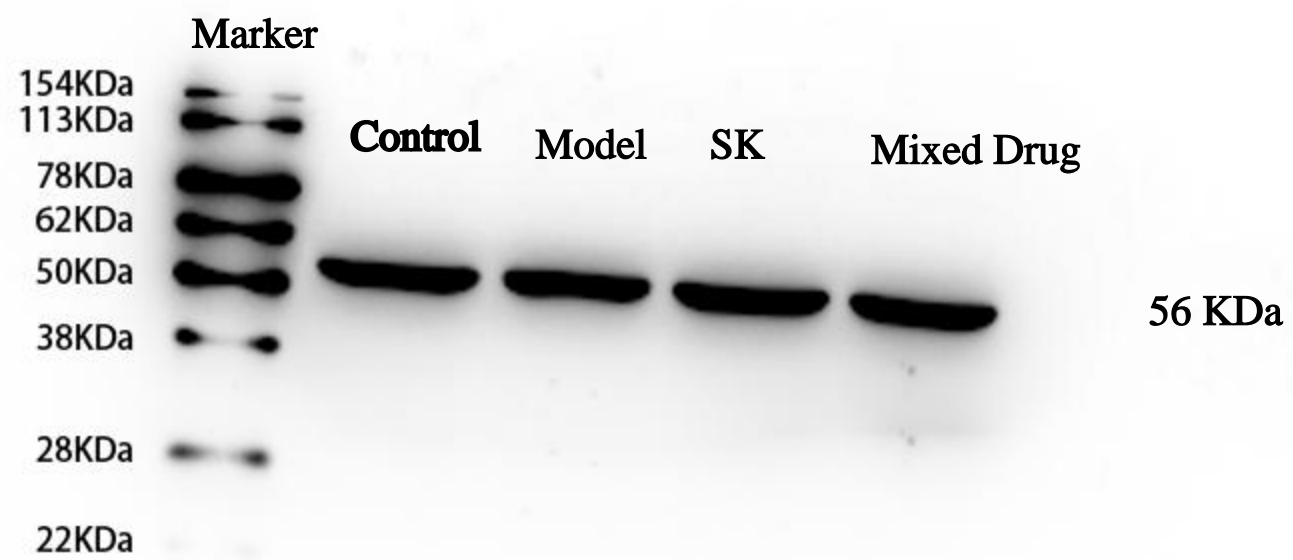

# GAPDH

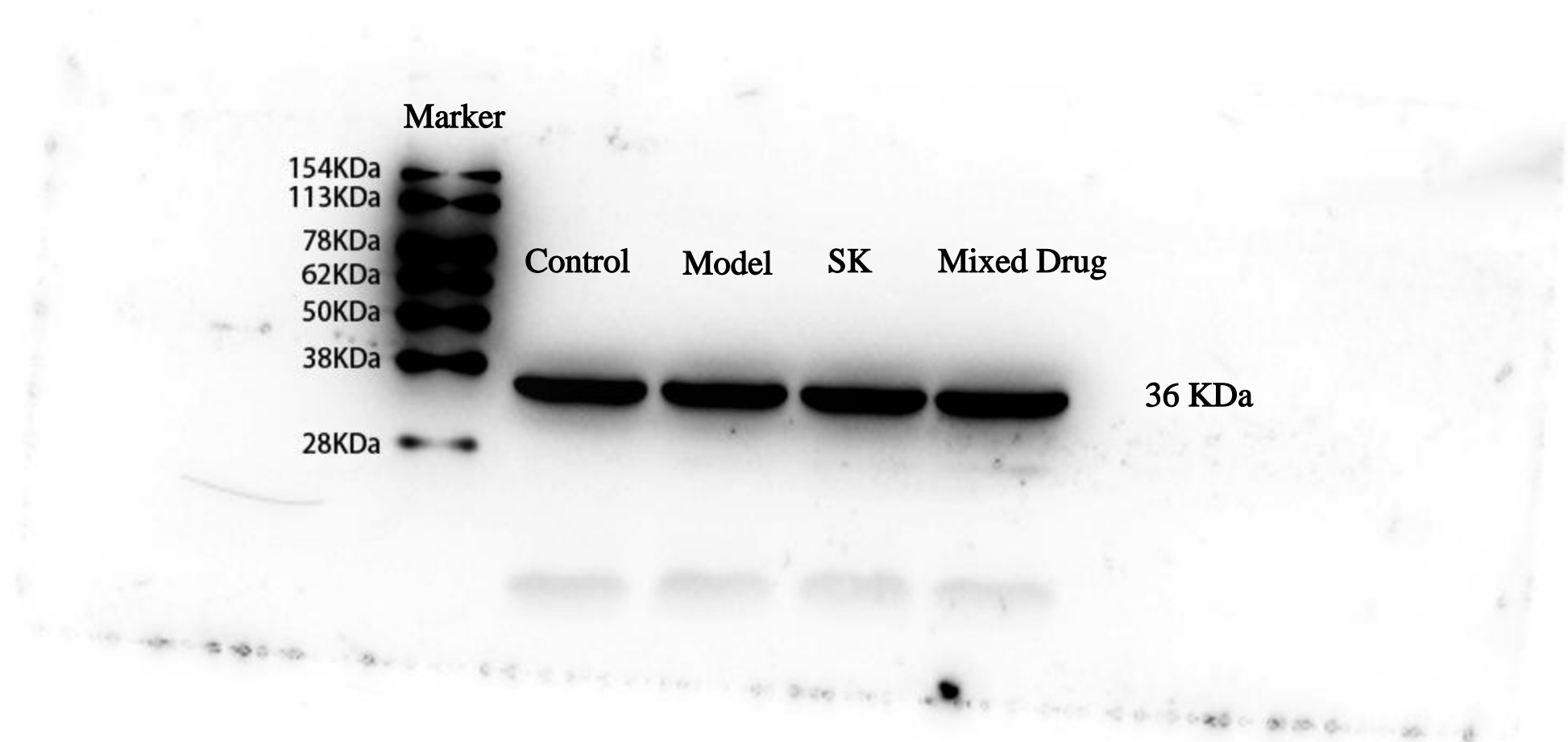

Supplement: S1 Raw images — (PDF) [file pone.0291621.s005.pdf]

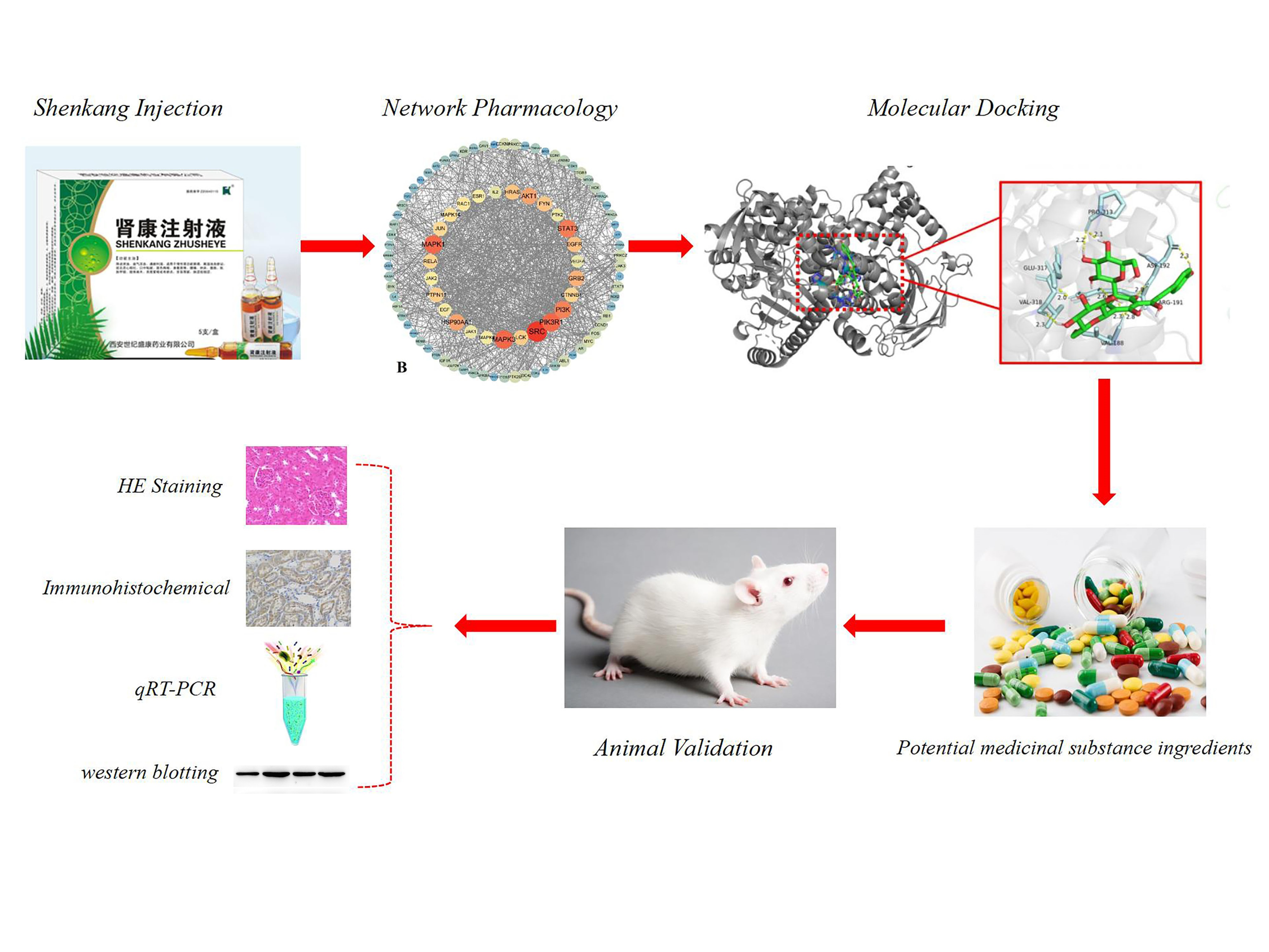

Supplement: S1 Graphical abstract — (TIF) [file pone.0291621.s006.tif]
